# Macrophage cell therapy enabled by interleukin-4 mRNA-loaded lipid nanoparticles to sustain a pro-reparative phenotype in inflammatory injuries

**DOI:** 10.1016/j.biomaterials.2025.123869

**Published:** 2025-11-25

**Authors:** Erin M. O’Brien, Tina Tylek, Hannah C. Geisler, Alvin J. Mukalel, Ricardo C. Whitaker, Samuel Sung, Benjamin I. Binder-Markey, Drew Weissman, Michael J. Mitchell, Kara L. Spiller

**Affiliations:** a School of Biomedical Engineering, Science, and Health Systems, Drexel University, Philadelphia, PA, USA; b Department of Bioengineering, School of Engineering and Applied Science, University of Pennsylvania, Philadelphia, PA, USA; c Department of Physical Therapy and Rehabilitation Sciences, Drexel University, Philadelphia, PA, USA; d Perelman School of Medicine, University of Pennsylvania, Philadelphia, PA, USA

## Abstract

The use of macrophage cell therapies is limited by their tendency to change phenotype in response to external cues in situ. Here we demonstrate that an optimized lipid nanoparticle (LNP) formulation effectively delivers IL4 mRNA to human and murine primary macrophages, resulting in rapid transfection, IL-4 secretion, and reparative phenotype modulation. In a model of murine volumetric muscle loss, adoptively transferred macrophages pre-treated with IL4-LNPs maintained a reparative phenotype for at least one week, despite the inflammatory injury microenvironment. IL4-LNP-treated macrophages also promoted a reparative phenotype in endogenous macrophages and supported muscle repair outcomes, including increased vascularization, fiber size distribution, and remodeling of the scaffold. T cell subtype in the muscle or the draining lymph node was not affected. The novel strategy established here may facilitate the control and use of macrophage cell therapies for other applications in regenerative medicine.

## 1. Introduction

Dysfunctional tissue repair is common in many conditions, including aging, diabetes, and catastrophic injuries. The resulting complications vary, from delayed wound closure and infection to fibrosis and loss of function. As key directors of the immune response, innate immune cells called macrophages are responsible for orchestrating a pro-regenerative environment within damaged tissue, such as skin and muscle [1–4]. By encompassing a wide spectrum of phenotypes, macrophages perform an equally broad range of functions congruous with the phase of healing. In early stages, monocytes infiltrate the site of injury and differentiate into pro-inflammatory macrophages, which clear bacteria, apoptotic cells, and tissue debris, and initiate angiogenesis [5–9]. Subsequently, reparative macrophages dominate the milieu to promote ECM deposition by fibroblasts and stabilize nascent vasculature [9–11]. Reparative macrophages can emerge in multiple ways: proliferation of existing reparative macrophages, recruitment and differentiation of circulating monocytes, or phenotypic switching of pro-inflammatory macrophages [7,12–17]. In studies of skin and muscle repair, macrophages activated with IL-4 (sometimes in combination with IL-13) in particular were crucial for healing, as they were responsible for proper collagen fibril assembly, myotube formation, and remodeling [7,11,18,19]. However, in dysfunctional repair, macrophage phenotype is dysregulated, resulting in chronic inflammation and delayed healing [20–26]. Studies in which macrophages are depleted in multiple phases of healing have demonstrated that functionality of both pro-inflammatory and reparative macrophages are required for complete tissue repair, thereby making macrophages attractive therapeutic targets [1,3,7]. Indeed, many regenerative medicine strategies have been proposed to promote tissue repair by modulating macrophage behavior (Refs. [27–30] and reviewed in Refs. [31,32]). However, it is still not clear how to correct dysfunctional macrophage behavior for improved tissue repair, because understanding of specific macrophage phenotypes and strategies to control them are lacking. For example, one recent study of volumetric muscle loss (VML) in mice showed that fibrotic muscle repair could be traced to macrophages that were not hyper-inflammatory, but insufficiently reparative [26], suggesting traditional anti-inflammatory approaches may actually hinder repair by dampening the inflammatory response required for healthy repair, while therapies that can promote a reparative macrophage phenotype would improve outcomes. These findings align with a growing body of literature that points to the necessity of a robust, but not prolonged, early inflammatory response for normal tissue repair [5,7,8,33].

Inhibited tissue repair frequently coincides with low macrophage numbers and dysfunctional macrophage behavior, both of which are major roadblocks for macrophage-targeting treatments [26,34,35]. Immune cell therapy is a quickly growing field of immune engineering, with notable successes in the use of chimeric antigen receptor (CAR) T cells in the treatment of blood cancers. More recently, CAR macrophages have been proposed for the treatment of solid tumors because of their superior ability to infiltrate dense tissues [36]. Given the critical role for macrophages in regulating tissue repair, there is major potential in expanding the application of macrophage cell therapy to regenerative medicine by delivering macrophages to non-healing injuries to promote tissue repair [37]. Several studies have shown that the adoptive transfer of macrophages can improve muscle vascularization and repair [38,39]. Adoptive transfer of macrophages to promote tissue repair would provide a two-pronged approach to address dysfunctional healing by 1) replacing macrophages lost through injury or lack of recruitment, and 2) exploiting the regulatory nature of macrophages to normalize the injury milieu, including host macrophages.

**Fig. 1.**
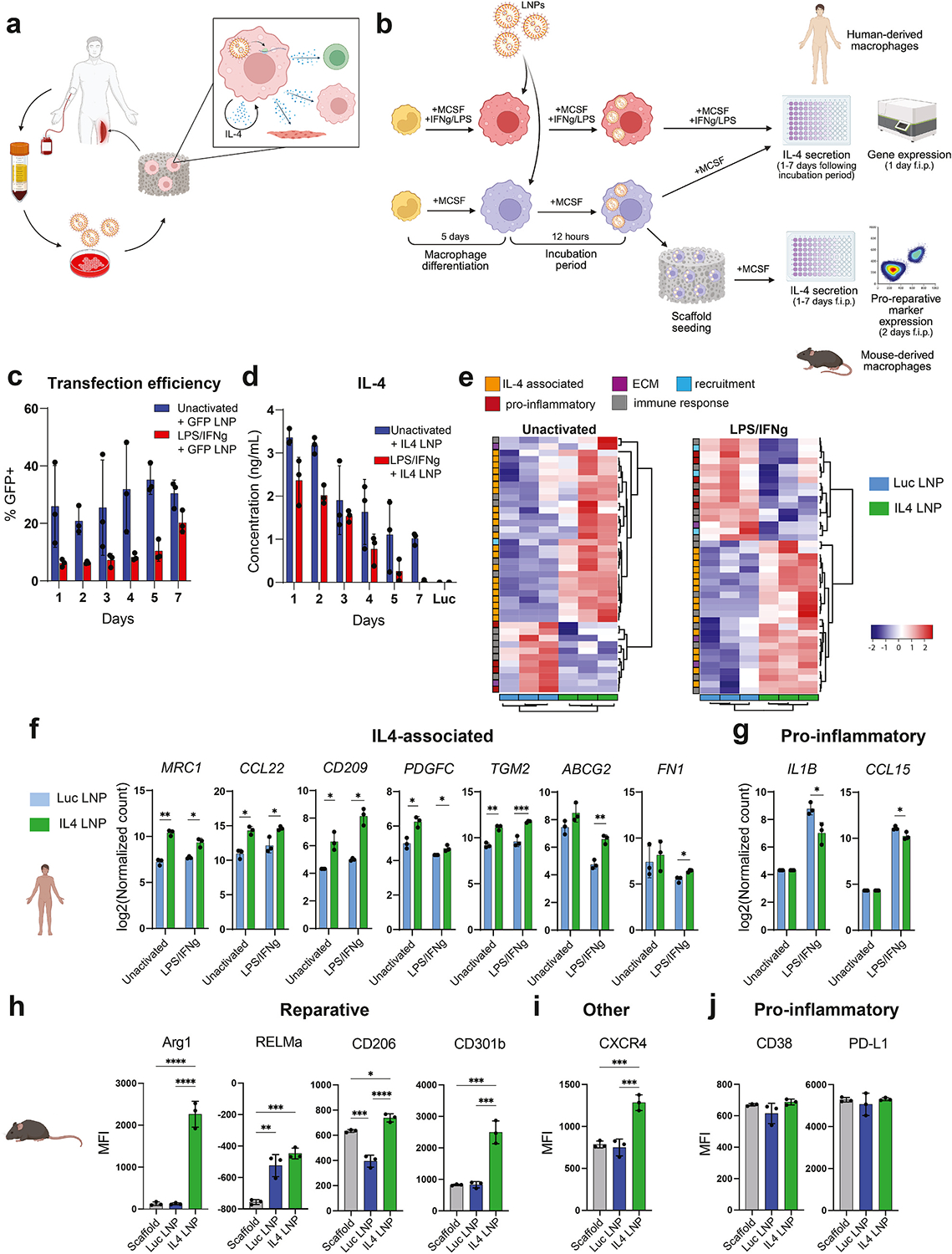
Effects of IL4-LNPs on primary macrophages in vitro. (**a**) Proposed biomaterial-mediated macrophage cell therapy strategy. Circulating monocytes are exogenously differentiated into macrophages, treated with IL4-mRNA-loaded LNPs, then seeded onto a porous biomaterial scaffold and implanted into the site of injury. As LNPs degrade inside the cell, mRNA is released and translated, resulting in IL-4 secretion. Secreted IL-4 promotes a reparative phenotype in endogenous and implanted macrophages, and also regulates neighboring immune cells and regenerating myofibers. (**b**) In vitro experimental design. Peripheral blood-derived human monocytes or bone marrow-derived murine cells are differentiated into macrophages, then treated with mRNA-LNPs for 12 h. Conditioned media for ELISA and cells for nanoString gene expression or flow cytometry were collected 1–7 days following the incubation period. (**c**) Transfection efficiency of macrophages with GFP mRNA. (**d**) Unactivated and pro-inflammatory human macrophage secretion of IL-4 one week following treatment with IL4-LNPs. (**e**) Hierarchical clustering of top 40 genes with lowest p-values expressed by unactivated or pro-inflammatory macrophages 24 h following treatment with Luc-LNPs or IL4-LNPs. Each column represents one human donor. (**f**) Human unactivated and pro-inflammatory macrophage expression of differentially expressed IL4-associated genes 24 h following treatment with Luc-LNPs or IL4-LNPs. Data are represented as mean ± SD. (**c-e**) Multiple t-tests with Holm-Sidak adjustment, n = 3 donors, *p_adj_ < 0.05. (**g**) Human unactivated and pro-inflammatory macrophage expression of differentially expressed pro-inflammatory genes 24 h following treatment with Luc-LNPs or IL4-LNPs. (**h-j**) Murine unactivated macrophage expression of (h) reparative, (i) angiogenic, or (j) pro-inflammatory markers 48 h following treatment with Luc-LNPs or IL4-LNPs and seeding on Surgifoam scaffolds. Data are represented as mean ± SD. One-way ANOVA with Tukey’s post-hoc, n = 3 experimental replicates, *p < 0.05.

Despite the demonstrated promise of macrophage cell therapy, one major obstacle limiting its potential is the extreme sensitivity of macrophages to external cues. While this characteristic is typically a necessary component of macrophage function, it presents an issue for cell therapy strategies. Cell therapies administered to non-healing injuries, which are chronically inflamed, require macrophages to exhibit and maintain a reparative phenotype in order to resolve inflammation and promote repair. Unless some method of maintaining phenotype is incorporated, even exogenously polarized macrophages will become more inflammatory in response to the microenvironment [37,40,41]. Ideally, such a method would be intrinsically integrated within the macrophages themselves to limit external interference or separation from the administered macrophages or treatment site. A potential route for phenotype modulation is the use of lipid nanoparticles (LNPs), which have been shown to effectively deliver mRNA to immune cells to generate chimeric antigen receptor (CAR) T cells [42–44]. However, most LNP formulations are immunogenic and trigger an inflammatory response – a desirable trait for anti-tumor therapies, but not for maintaining a reparative macrophage phenotype in inflammatory injuries. Additionally, macrophages are historically difficult to transfect compared to other cell types [45]. Recent work from Mukalel et al. identified numerous LNP formulations in pursuit of CAR-macrophage engineering that incorporated an oxidized lipid structure to increase uptake by macrophages with minimal toxicity [46]. A library of LNPs was screened to select a formulation that maximized delivery of the gene encoding luciferase into human THP1-derived macrophages while minimizing toxicity, then optimized using an orthogonal design of experiments (DoE) approach to yield an optimized formulation with further improved transfection efficiency. Optimization of LNP formulation parameters including lipid structure and excipient ratios addressed the primary issues with LNP-mediated mRNA delivery to macrophages, achieving successful transfection of macrophages with minimal inflammatory activation, although the mechanism behind the minimal inflammatory activation is not yet known. We therefore sought to incorporate this formulation into a cell therapy strategy in which monocytes derived from patient peripheral blood could be exogenously differentiated into macrophages and treated with IL4-mRNA-loaded LNPs, so that they would secrete IL-4 (normally secreted by T cells, eosinophils, and basophils, but not macrophages [47]) to maintain a reparative phenotype while also promoting reparative outcomes in host macrophages and regenerating muscle fibers (Fig. 1a).

First, we assessed the ability of the optimized LNP platform to deliver mRNA encoding the gene for IL-4 and its effects on macrophage phenotype in vitro using primary murine and human macrophages. Then, we used a murine model of VML in the quadriceps to test the IL-4 engineered macrophages’ ability to resist pro-inflammatory repolarization and promote tissue repair. Together, these studies demonstrate proof-of-concept of a novel macrophage cell therapy strategy that enables macrophages to maintain a desired phenotype in vivo in spite of their environment.

## 2. Results

### 2.1. IL4-LNPs transfect primary macrophages and promote a reparative phenotype

First, we evaluated the ability of LNPs encapsulating mRNA encoding IL-4 (IL4-LNPs) to transfect macrophages, induce IL-4 secretion, and promote a reparative phenotype in vitro (Fig. 1b). To first measure transfection efficiency, we treated unactivated or pro-inflammatory (activated with lipopolysaccharide and interferon-gamma) primary human macrophages with LNPs containing mRNA encoding for green fluorescent protein (GFP), then used flow cytometry to quantify the percentage of GFP + macrophages over one week. Unactivated macrophages ranged from about 20 to 50 % positive (Fig. 1c). Pro-inflammatory macrophages, on the other hand, were only about 6 % positive at day 1, but this increased to 20 % by day 7.

We then treated unactivated or pro-inflammatory macrophages with IL4-LNPs, or luciferase-encoding LNPs (Luc-LNPs) as a negative control, at a dose of 500 ng per 50,000 cells. After 12 h, excess LNPs were washed away, then IL-4 secretion was measured over one week in vitro using enzyme linked immunosorbent assay (ELISA). Both unactivated and pro-inflammatory macrophages treated with IL4-LNPs were rapidly transfected, secreting high amounts of IL-4 within 24 h after LNP treatment (Fig. 1d). IL-4 secretion continued at detectable levels for at least a week, with higher levels of secretion for unactivated macrophages compared to pro-inflammatory macrophages (Fig. 1d). Unactivated murine macrophages were tested at lower doses to maximize viability following treatment (Supplementary Fig. 1a), and were also successfully transfected, secreting IL-4 at detectable doses for at least a week (Supplementary Fig. 1b). Untreated controls or Luc-LNP-treated macrophages did not secrete detectable levels of IL-4 at any time point.

To test whether the secreted IL-4 could promote a reparative phenotype in human macrophages, we used nanoString multiplex gene expression analysis to measure expression of a custom panel of 200 markers (Supplementary Table 1), 24 h after the end of the 12-h LNP treatment period. Hierarchical clustering of the top 40 genes in each group based on the lowest p-value showed clear differences between samples incubated with Luc-LNPs versus IL4-LNPs (Fig. 1e). Several genes associated with the IL-4-mediated reparative macrophage phenotype were significantly upregulated in both unactivated and pro-inflammatory macrophages treated with IL4-LNPs (Fig. 1f), demonstrating IL-4-mediated polarization in just one day following LNP treatment, even when macrophages were first polarized to a pro-inflammatory phenotype. IL4-LNPs also caused human pro-inflammatory macrophages to downregulate inflammatory markers *IL1B* and *CCL15* (Fig. 1g). Given that these macrophages were continuously cultured in IFNg/LPS-supplemented media, this finding demonstrates the capability of IL4-LNP-loaded macrophages to promote a reparative phenotype despite the presence of inflammatory stimuli. *ABCG2* and *FN1* were increased to a greater extent in macrophages that were first pro-inflammatory prior to IL4-LNP treatment, reflecting previous findings showing differences in macrophage responses to soluble IL-4 depending on their activation state [33]. Several additional differentially expressed genes (DEGs) were not strongly associated with IL-4 activation, suggesting potentially more complex effects on macrophage phenotype (Supplementary Fig. 2).

To measure polarization of murine macrophages while also testing a potential cell delivery vehicle, we seeded 1 million macrophages immediately following the LNP treatment period onto 4 mm diameter and 2 mm thick porous gelatin (Surgifoam) scaffolds, which we previously determined to have neutral effects on macrophages [48], then analyzed macrophage phenotype on the protein level by flow cytometry 48 h later. IL4-LNPs caused murine macrophages to upregulate reparative markers, particularly Arg1 and CD301b, as well as the angiogenesis-associated marker CXCR4 (Fig. 1h–i). Neither scaffold alone nor Luc-LNPs caused an increase in pro-inflammatory marker expression, suggesting neither would have significant unintended effects on macrophage phenotype (Fig. 1j).

### 2.2. IL4-LNP-treated macrophages maintain a reparative phenotype in vivo in a murine model of volumetric muscle loss

**Fig. 2.**
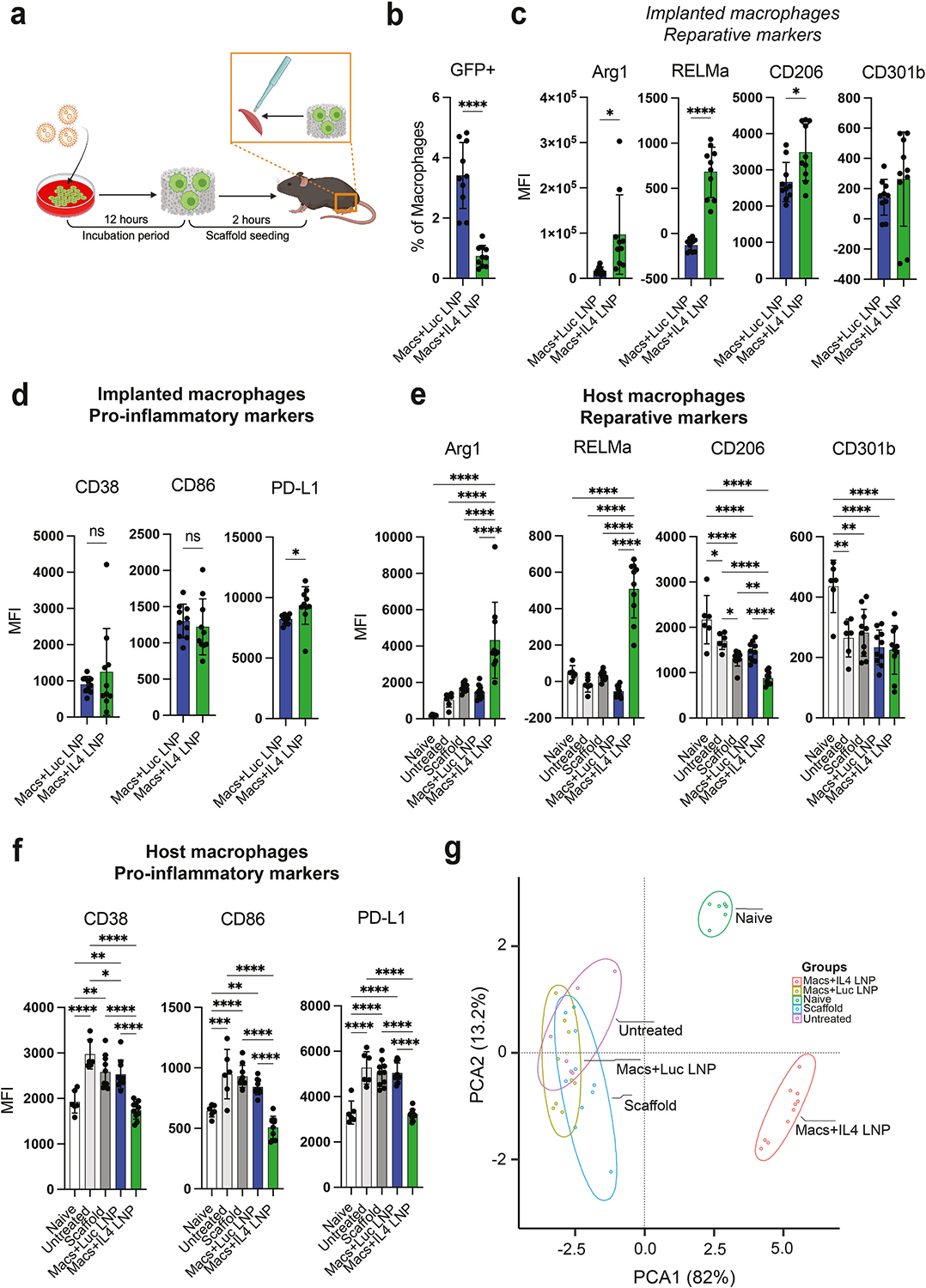
Phenotype of adoptively transferred and endogenous macrophages in a murine model of volumetric muscle loss treated with IL4-LNP cell therapy. (**a**) In vivo experimental design. GFP + murine macrophages were treated with mRNA-LNPs for 12 h, then seeded on gelatin scaffolds for at least 2 h. Seeded scaffolds were implanted into 4 mm muscle defects in the quadriceps of wild-type mice. (**b**) Prevalence of implanted GFP + macrophages 7 days following injury. Data are represented as mean ± SD. Student’s t-test, n = 10 mice, *p < 0.05, **p < 0.01, ***p < 0.001, ****p < 0.0001. (**c-d**) Expression of reparative (c) and pro-inflammatory (d) markers by implanted macrophages treated with Luc-LNPs or IL4-LNPs one week following implantation in VML model. (**e-f**) Expression of reparative (e) and pro-inflammatory (f) markers by host macrophages one week following VML injury and treatment. One-way ANOVA with Tukey’s post-hoc, n = 6–10 mice. (**g**) Principal component analysis of pro-inflammatory and reparative marker expression by endogenous macrophages one week following VML injury and treatment.

To test the cell therapy strategy in vivo, we used a murine model of volumetric muscle loss (VML), in which a 4 mm biopsy punch was used to create a critical-sized defect in the quadriceps, which generates a pro-inflammatory environment characterized by dysregulated macrophage phenotype as early as 1 day after injury and leading to defective, fibrotic repair 28 days later [26,49,50] (Fig. 2a). First, we confirmed previous reports [41,51,52] that ex vivo-polarized macrophages do not maintain their phenotype in vivo by implanting macrophages, derived from GFP-transgenic mice, that had been pre-polarized with soluble IL-4. After 3 days, the entire muscle was explanted, and flow cytometry was used to characterize the implanted (GFP + Live/Dead-CD45+F4/80+) and endogenous (GFP-Live/Dead-CD45+F4/80+) macrophages. The implanted macrophages dramatically downregulated the reparative markers CD301b and CD206 and upregulated the pro-inflammatory marker CD38 compared to pre-implantation levels (Supplementary Fig. 3). Interestingly, the reparative marker Arg1 and the pro-inflammatory marker PD-L1 did not change compared to pre-implantation levels, showing that phenotype changes are complex and that interpretation of data can be influenced by the markers employed.

One million macrophages were treated with Luc-LNPs or IL4-LNPs and seeded onto 4 mm scaffolds, then placed inside the defect immediately following injury in male and female C57Bl/6 wild-type mice. After 7 days, the entire muscle was resected and processed for flow cytometry analysis of pro-inflammatory or reparative macrophage phenotype markers. In the IL4-LNP group compared to the Luc-LNP group, there were significantly fewer GFP + macrophages remaining when counted as a percentage of total macrophages (Fig. 2b), and as a total count (Supplementary Fig. 4). However, reparative markers were all upregulated in the IL4-LNP-treated macrophages compared to Luc-LNP-treated macrophages (Fig. 2c), indicating that expressed IL-4 promoted a reparative phenotype for at least one week. Despite the apparently low number of implanted macrophages relative to macrophages overall, the results were similar when presented as percent positive (Supplementary Fig. 5a). There were no differences in expression of pro-inflammatory markers between GFP + macrophages that had been treated with Luc-LNPs or IL4-encoding LNPs (Fig. 2d, Supplementary Fig. 5a), with the exception of slightly higher PD-L1 levels in the IL4-LNP group. There were no major differences in macrophage phenotype based on sex, with the exception of Arg1, which was slightly higher in female mouse macrophages in the Luc-LNP group (Supplementary Fig. 6a).

### 2.3. IL4-secreting macrophage cell therapy promotes a reparative phenotype in host macrophages

After confirming that transplanted macrophages that had been treated with IL4-LNPs maintained a reparative phenotype, we next wanted to determine whether they influenced the phenotype of host macrophages. At 7 days post-injury, host macrophages (GFP-LiveDead-CD45+F4/80+) from injured muscle were phenotyped for the same pro-inflammatory and reparative markers as implanted macrophages. Endogenous macrophages in the IL4-LNP group exhibited significantly higher expression of reparative markers Arg1 and RELMα compared to all other groups (Fig. 2e, Supplementary Fig. 5b). They expressed CD206 at lower levels than all other control groups, and CD301b expression was unaffected by treatment.

As anticipated, untreated VML injury caused significant upregulation of several pro-inflammatory markers in endogenous macrophages compared to naïve (uninjured) controls (Fig. 2f, Supplementary Fig. 5b). Implantation of the scaffold alone or scaffolds seeded with macrophages that had been treated with Luc-LNPs failed to mitigate this inflammation, but treatment with IL4-LNP-treated macrophages resulted in decreased pro-inflammatory marker expression to levels comparable to those of the naïve group. Interestingly, host macrophages in female mice expressed higher levels of CD38 and PD-L1 than those in male mice in untreated groups but not in groups treated with IL4-LNP-macrophages (Supplementary Fig. 6b). Principal component analysis (PCA) of endogenous macrophage MFI indicated a strong separation of the IL4-LNP group, while untreated, scaffold, and Luc-LNP groups all closely associated (Fig. 2g). PCA inclusive of adoptively transferred macrophages showed similar groupings, with implanted IL4-LNP-treated macrophages clustering near host macrophages in the same treatment group (Supplementary Fig. 7). On a gross level, the wet weight of injured muscle 7 days following injury did not significantly differ from the weight of the contralateral muscle, although they were lower (Supplementary Fig. 8). Altogether, these data suggest that IL4-LNP-treated macrophages promote a reparative phenotype in both the transplanted population and in neighboring host macrophages for at least one week post-implantation.

### 2.4. IL4-LNP macrophage cell therapy promotes muscle repair

**Fig. 3.**
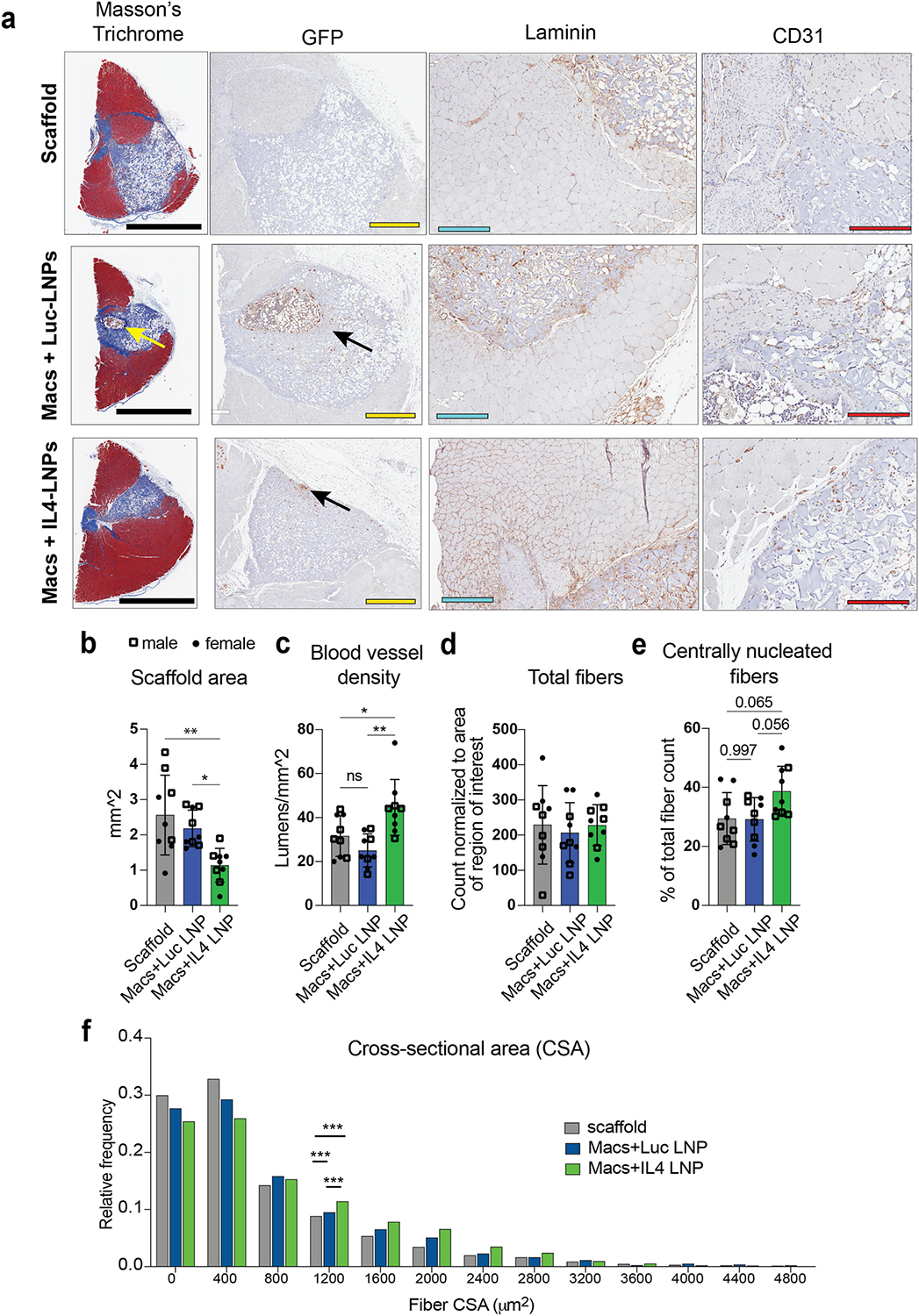
Histological analysis of muscle repair 28 days after injury. (**a**) Representative images of histology sections stained for Masson’s Trichrome, GFP, Laminin, CD31. Black scale bar = 2 mm. Yellow scale bar = 600 μm. Blue scale bar = 300 μm. Red scale bar = 200 μm. (**b**) Area of implant remaining at 28 days following VML injury and treatment. Data are represented as mean ± SD. (**c**) Density of CD31^+^ blood vessel lumens in vicinity of scaffolds. (**d**) Number centrally nucleated fibers identified via laminin staining in vicinity of scaffolds. (**e**) Proportion of total fibers that were centrally nucleated. (**b-e**) One-way ANOVA with Tukey’s post-hoc, n = 9 biological replicates, *p < 0.05, **p < 0.01. (**f**) Relative frequency distribution of cross sectional area (CSA) of fibers in the vinity of the scaffolds. Pairwise Kolmogorov–Smirnov distribution tests were performed between each condition (n = 9 biological replicates, ***p < 0.001).

Having established that IL4-LNP-treated macrophages promote a reparative phenotype in endogenous macrophages, we next sought to determine the effects of the cell therapy on muscle repair outcomes. At 28 days following VML injury and treatment, the affected muscles were sectioned and stained for collagen content (Masson’s Trichrome), the presence of transplanted macrophages (anti-GFP), vasculature (CD31), and muscle fibers (laminin) (Fig. 3a). Masson’s Trichrome staining clearly delineated muscle tissue versus the implanted gelatin scaffold. The cross-sectional area of scaffold remaining in the injury was lower in the IL4-LNP-treated macrophage group compared to Luc-LNP-treated and scaffold alone (Fig. 3b, Supplementary Fig. 9–10). Vascularization, as measured by density of CD31^+^ blood vessels in the region surrounding the implant, was also higher in the IL4-LNP group (Fig. 3c). While the total number of fibers in the vicinity of the implant did not differ between groups (Fig. 3d), the proportion of centrally-nucleated muscle fibers, indicative of those actively regenerating [53], in the region surrounding the implant was higher in the IL4-LNP group, although this result fell short of statistical significance (p = 0.056) (Fig. 3e). Finally, the distribution of cross sectional area (CSA) of muscle fibers in the region surrounding the implants significantly differed between all three groups, with muscles treated with IL4-LNP-macrophages showing a distribution of larger fibers compared to the other control groups (Fig. 3f). In particular, there was with a significantly higher proportion of larger fibers (>1200 μm^2^) in the IL4-LNP-macrophage group compared to the other control groups, suggesting actively regenerating fibers. Interestingly, the Luc-LNP-macrophage group also had a higher proportion of fibers larger than 1200 μm^2^ compared to the scaffold control group, but less than the IL4-LNP-macrophage group. Altogether, these results point to increased scaffold remodeling, vascularization, and repair, in muscle treated with IL4-LNP-treated macrophages.

At 7 days, and surprisingly even 28 days after implantation, some GFP + macrophages remained in the scaffold in both the IL4-LNP and Luc-LNP group (Fig. 3a, Supplementary Figs. 11–12). However, there were more GFP + macrophages in the Luc-LNP group, and they appeared to congregate in areas of the scaffold coinciding with high levels of collagen. Additionally, some GFP + cells could be found outside of the scaffolds, indicating migration into the muscle as early as one week following implantation (Supplementary Figs. 11–12). Collectively, these data show that treatment with IL4-LNP-loaded macrophages promotes muscle repair in murine VML.

### 2.5. IL4-LNP macrophage cell therapy modulates the local immune response

**Fig. 4.**
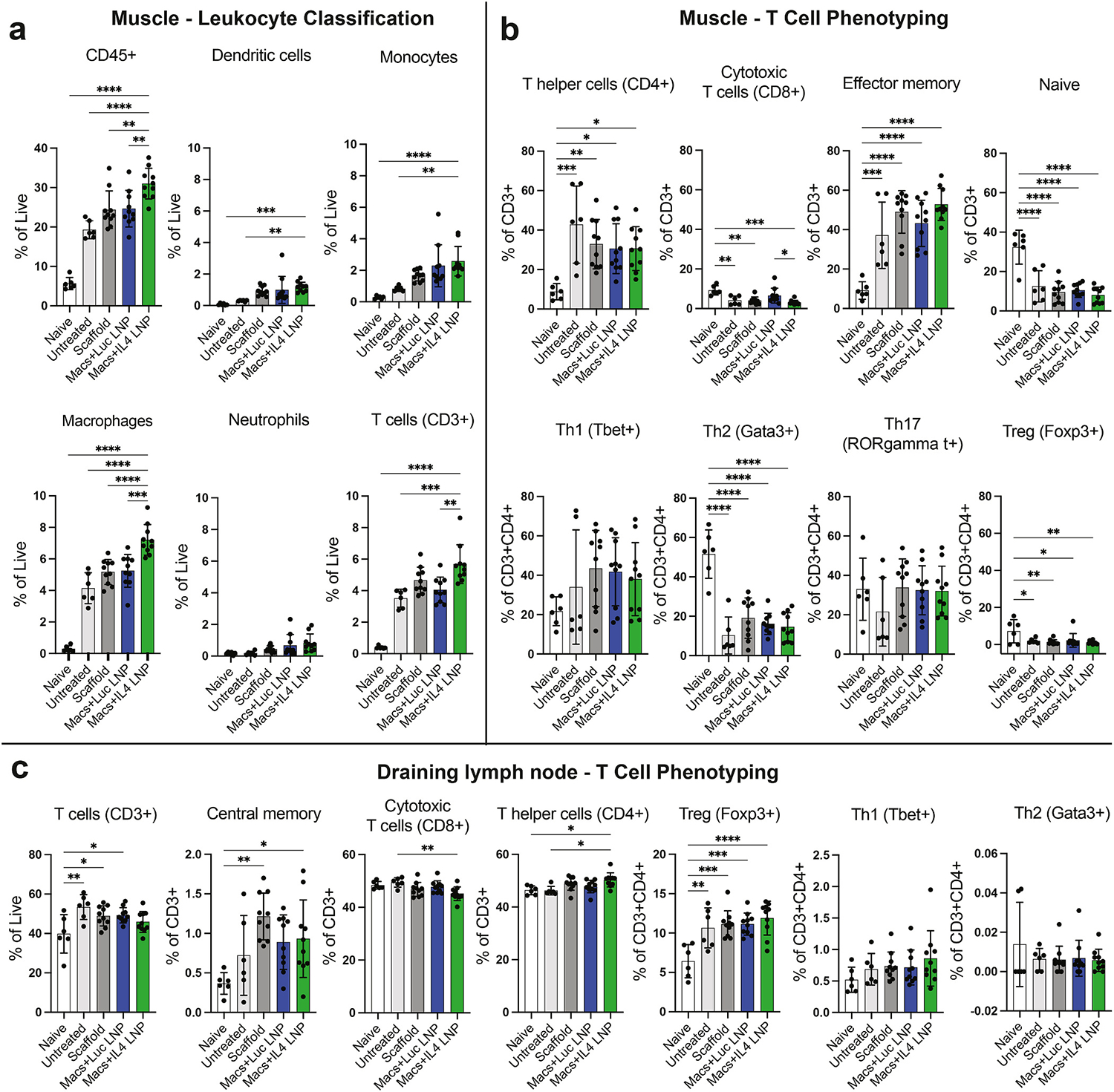
Characterization of immune response to IL4-LNP cell therapy. (**a**) Classification of muscle-infiltrating leukocytes one week following VML injury and treatment. Data are represented as mean ± SD. One-way ANOVA with Tukey’s post-hoc, n = 6–10 mice, *p < 0.05, **p < 0.01, ***p < 0.001, ****p < 0.0001. Some significance not shown for visual clarity; complete graphs shown in Supplementary Fig. 16 (**b**) Phenotyping of muscle-infiltrating T cells one week following VML injury and treatment. (**c**) Phenotyping of T cells in draining inguinal lymph node one week following VML injury and treatment.

To evaluate the effects of the IL-4-LNP-macrophage cell therapy on the local immune response to volumetric muscle loss, we identified leukocytes infiltrating the injured muscle, and characterized phenotypes of T cells both within the muscle and in the draining inguinal lymph node, at one week post-implantation. Compared to naïve muscle, untreated VML injury induced a significant increase in CD45^+^ leukocytes as a percentage of all live cells (Fig. 4a). Treatment with IL4-LNP-treated macrophages further increased CD45^+^ cell infiltration, indicating a boost in immune cell recruitment, whereas the scaffold alone or scaffold seeded with Luc-LNP-treated macrophages increased total CD45^+^ cells only slightly and not significantly compared to untreated. Likewise, the IL4-LNP group caused a significant increase in dendritic cells (DCs), monocytes, macrophages, and T cells. There was also a minor increase in neutrophil presence that was not statistically significant. Female mice exhibited slightly increased infiltration of DCs, monocytes, and neutrophils compared to male mice, though only DC infiltration in the IL4-LNP group showed statistical significance (Supplementary Fig. 13).

Within the muscle, VML injury caused an increase in total CD3^+^ T cells, which was further increased with IL4-LNP-treated macrophages. However, T cell phenotype markers were not strongly influenced by treatment, with no differences in markers of helper T cells, cytotoxic T cells, effector memory T cells, naïve T cells, Th1, Th2, Th17, or regulatory T cells, with the exception of significantly more cytotoxic T cells in the Luc-LNP group compared to the IL4-LNP group (Fig. 4b). Although there were no statistically significant differences between T cells in female versus male mice, female mice did appear to have potentially higher levels of CD8^+^ and Th1 cells, while male mice exhibited higher levels of naïve and regulatory T cells (Supplementary Fig. 14).

We also phenotyped T cells one week post-injury within the draining lymph node proximal to the injured muscle to look for any systemic effects of IL4-LNP macrophage treatment. Compared to lymph nodes from naïve mice, VML-injured mice exhibited an increase in all CD3^+^ T cells, though this was not significant in the IL4-LNP group (Fig. 4c). The IL4-LNP group significantly increased the presence of CD4^+^ T helper cells and decreased the presence of CD8^+^ cytotoxic T cells compared to untreated mice. Interestingly, all VML-injured groups had significantly more Tregs within the lymph node compared to naïve animals, converse to the profile of muscle-infiltrating T cells. There were no obvious differences in T cell phenotype between female and male mice; male mice had slightly higher levels of naïve T cells and Th2 cells, but these numbers were extremely low overall (Supplementary Fig. 15). Collectively, these data demonstrate a boosted local immune response one week after adoptive transfer of IL4-LNP-treated macrophages, with some effects on T cell phenotype locally and systemically.

## 3. Discussion

In this study, we have demonstrated proof of concept of a novel macrophage cell therapy strategy in which IL-4 mRNA-loaded LNPs modulate macrophage phenotype. In a model of inflamed and fibrotic VML, IL4-LNP-treated macrophages maintained an IL4-mediated reparative phenotype, promoted a similar phenotype in endogenous macrophages, modulated the surrounding immune microenvironment, and stimulated processes associated with muscle repair. To our knowledge, this is the first demonstration of an LNP-controlled macrophage cell therapy towards tissue repair. Further, enabling macrophages to overcome their acute sensitivity to environmental factors is a crucial step towards developing translational macrophage cell therapies.

Several attempts have been made within the field of regenerative medicine to develop macrophage cell therapies with phenotypic control, both with and without the support of biomaterials. In one such study, Risser et al. co-administered IL-4-activated macrophages and PLGA microparticles loaded with IL-4 to a murine model of hindlimb ischemia [39]. Although this treatment improved angiogenesis outcomes, recovery of adoptively transferred macrophages was low, raising questions concerning the role of any phenotype maintenance in the observed results, as well as the limitations of administering cells via direct injection [54,55]. Moreover, no strategy was employed to ensure that the transplanted macrophages would co-localize with the IL-4-releasing microparticles. In this study we overcame these challenges by utilizing a biomaterial scaffold carrier to maintain macrophages within the site of injury, and by engineering the macrophages themselves to secrete IL-4, reasoning that they would act in an autocrine and paracrine manner. In the absence of any method to maintain phenotype post administration, murine macrophages pre-treated with soluble IL-4 shifted to a pro-inflammatory phenotype within three days after implantation in a VML injury, confirming previous reports [56]. The results from these studies emphasize the importance of both an intrinsic method of maintaining a reparative phenotype, as well as a cell delivery vehicle that can anchor macrophages to the treatment site. Here, we show that IL4-LNP-treated macrophages successfully maintained a reparative phenotype for at least one week after implantation in VML injuries. The gelatin scaffold used here did appear to maintain the presence of implanted macrophages through day 28 without influencing phenotype, but further investigation is required to determine the ideal cell delivery vehicle. These results suggest the potential for long-term immunomodulation within the injury environment.

The main innovation of this cell therapy strategy relies upon transfecting macrophages with mRNA encoding for the gene for IL4, a cytokine not normally secreted by macrophages, to allow the population to self-regulate to maintain a reparative phenotype and also influence host macrophage phenotype. mRNA-loaded LNPs have recently begun to make headway in the realm of cell therapy, primarily in CAR T cells and other anti-tumor strategies [57]. More traditional gene editing techniques like viral transfection have been shown to be pro-inflammatory to macrophages [56], rendering them unsuitable in the context of tissue repair. The use of viral vectors is also disadvantageous from a manufacturing perspective because of the difficulty to scale up manufacturing, high cost of goods, and the need for complex quality control measures to ensure that no live viral vector is transferred into the patient. Compared to viral vector-based transduction, LNP-mediated delivery of mRNA is less immunogenic and poses a lower risk of mutagenesis [43]. However, very few mRNA-LNP-enabled cell therapies have been developed towards tissue repair, as LNPs in general are still inherently inflammatory, and those that do exist are typically stem cell-rather than immune cell-based [58,59], likely because immune cells tend to evade transfection. Here we used recently developed formulations that were specifically designed to transfect macrophages with minimal inflammatory response [60]. Although these LNPs were originally designed for use in CAR-macrophage engineering, we demonstrate successful transfection of human and murine macrophages with IL-4 mRNA and subsequent control over reparative macrophage phenotype. The approach presented here sought to address the problems stalling further development of pro-reparative macrophage cell therapies, as their ease of extraction from peripheral blood and their role as master regulators of other cell types make macrophages appealing targets. By using a novel non-inflammatory, macrophage-directed LNP formulation, we have demonstrated for the first time an mRNA-LNP-mediated cell therapy that successfully enables macrophages to promote tissue repair in an inflammatory injury.

An interesting observation from this study was that transplanted GFP + macrophages were found in the defect site after 28 days in vivo, although IL4-LNP-treated macrophages were present in much lower numbers compared to Luc-LNP-treated macrophages. The Luc-LNP-treated macrophages also appeared to congregate in areas associated with increased collagen content, suggesting that apoptosis or migration of implanted macrophages, potentially mediated by IL-4, may be a healthy component of cell therapy-mediated repair. Although interference by the gelatin (collagen-derived) scaffold prevented direct quantification of collagen, these high-collagen areas could be indicative of fibrosis or foreign body giant cells (FBGCs), which are both characterized by collagen deposition and are frequently attributed to dysfunctional macrophage behavior. The role of IL-4-activated macrophages in particular as drivers of fibrosis and FBGC formation has long been a controversial topic [61–64], but the lack of these collagen-concentrated areas in the IL4-LNP-macrophage-seeded scaffolds challenges this idea. In fact, the decreased area of remaining scaffold in the IL4-LNP group implicates IL4-activated macrophages in active degradation of the implant. Previously we showed that macrophages polarized with IL-4 in vitro upregulated secretion of MMP12, which degrades collagen [65]. On the other hand, secreted factors from the macrophages, including IL-4, could also induce other cell types in the vicinity, such as fibroblasts, to increase collagen degradation. Future studies are required to investigate this mechanism in detail since it has major implications for tissue repair and regeneration.

Although transplanted IL4-LNP-loaded macrophages upregulated all the reparative phenotype markers measured – Arg1, RELMα, CD206, and CD301b – endogenous macrophages only upregulated Arg1 and RELMα. Within this group, expression of CD206 and CD301b decreased or remained at the same levels of the injured control groups, contrary to the expectation that IL4-polarized macrophages would upregulate all of the typical pro-reparative markers. However, the complexity of macrophage phenotype, particularly in vivo, is becoming increasingly appreciated in the field of immune engineering. It is possible that the discrepancy between implanted and host macrophages may be related to the fact that the transplanted macrophages were derived from unactivated macrophages, while endogenous macrophages within the injury were very likely pro-inflammatory prior to IL-4 exposure. Previous work in human macrophages showed that pro-inflammatory activation changes their subsequent response to IL-4, generating a phenotype with a unique gene and protein signature that is still pro-reparative (“M2-like”) but with potentially enhanced reparative functionalities [33]. Given that inflammation is a crucial phase in complete tissue repair, both types of reparative macrophages may be necessary to work in a complementary manner. Therefore, adoptive transfer of IL4-LNP-loaded unactivated macrophages into an inflammatory injury may be an ideal approach to ensure functional diversity.

In contrast to the significant phenotype changes seen in endogenous macrophages, T cells in both the local injury site and the draining lymph node exhibited little to no changes in phenotype in response to IL4-LNP-mediated cell therapy. The lack of Th2 cells in particular was interesting, as IL-4 has long been associated with this T cell subtype, usually in the context of allergic response. Taken together with the limited changes in the lymph node, these findings suggest that the effects of this macrophage cell therapy are primarily localized to macrophages within the muscle. However, the increased infiltration of leukocytes, including macrophages and T cells, as well as the observed migration of implanted macrophages out of the delivery scaffold, warrants deeper investigation into the systemic response to the treatment.

Stratification of outcomes by sex revealed few significant differences between male and female mice but overall points to a slightly more pro-inflammatory injury microenvironment in female mice. Given that VML was induced with a 4 mm biopsy punch in all mice, it should be noted that this created a proportionally larger injury in the female mice, which would reasonably result in greater inflammation [26]. Furthermore, these findings align with a growing body of literature describing sex-based divergence in the inflammatory response to injury [66–68]. Overall, the findings of this study emphasize the importance of considering sex differences in the design and testing of pro-reparative immunotherapies.

This study establishes proof-of-concept that IL4-encoding LNPs can enable adoptively transferred macrophages to successfully overcome an inflammatory injury environment in vivo and maintain the desired phenotype. Using LNPs to deliver mRNA to macrophages, we developed a cell therapy strategy in which macrophages secrete IL-4 to both self-regulate phenotype as well as act as an IL-4 delivery system to neighboring cells. The transfection efficiencies reported in our study are on the same order as has been reported in the literature for LNPs designed to deliver genes encoding chimeric antigen receptors (CAR) to primary macrophages [69]. Because IL4 is secreted, transfected macrophages can influence the phenotype of surrounding macrophages, so the relatively low transfection efficiency would be expected to be less problematic than it might be for the development of CAR-macrophages. The IL4-LNP-engineered macrophages promoted a pro-reparative milieu for at least one week in VML injury, leading to improved tissue repair. The main novelty of these findings lies in the cell therapy platform, which addresses the longstanding challenge of macrophage phenotype changes in response to the implantation microenvironment. To our knowledge, this is the first instance in regenerative medicine in which mRNA-loaded LNPs have been used to modulate macrophages in a cell therapy application. The implications of these results may even reach beyond regenerative medicine, enabling the use of macrophages in cell therapies for any number of applications.

Although pro-reparative macrophages are frequently modeled through cytokine activation with both IL-4 and IL-13, we opted to transfect macrophages solely with IL4 mRNA. Others have shown that there is no significant difference between macrophages polarized by IL-4 alone or IL-13 alone [70]. Thus, we hypothesized that IL-4 alone would be sufficient to promote a pro-reparative phenotype in host and implanted macrophages. Additionally, delivering one type of mRNA allowed for a simpler experimental design for this initial proof-of-concept study, but future studies could easily include additional genes. One limitation of this study was that we were unable to specifically determine if the observed pro-reparative effects of the IL4-LNP-macrophages resulted from actions of the transplanted macrophages, their effects on host macrophages, or both. Additionally, it is difficult to discern if the effects of the IL4-LNP-treated macrophages on the host macrophages were mediated by secreted IL4 or other signaling factors secreted by the transplanted macrophages, such as PDGF-BB or CCL22 [33], which could be a crucial aspect of the cell therapy treatment. Future studies should be directed at understanding these mechanisms, in particular because host macrophages in VML injuries are known to exhibit dysfunctional reparative behavior [26], which may have been remedied by the cell therapy used here. Additionally, we measured some aspects of tissue repair, including vascularization, remodeling of the scaffold, the proportion of centrally-nucleated muscle fibers, and increased muscle fiber cross sectional area. However, functional analysis, oxidative stress, and other measures of functional muscle regeneration were not measured. Our main goal was to determine whether IL4-LNP treatment would be sufficient to induce macrophages to maintain a pro-reparative phenotype in spite of an acutely inflammatory microenvironment, and whether this would have long term effects on muscle repair. We were able to show that both implanted and endogenous macrophages were significantly less inflammatory and more reparative when implanted macrophages were pre-treated with IL4-LNPs, demonstrating proof-of-concept of the cell therapy platform. Future studies are required to improve the therapeutic effects of the treatment and measure its effects on muscle regeneration, which will likely depend on determining the IL-4 release kinetics in vivo, as well as optimizing both the dose of LNPs used to treat the macrophages and the number of macrophages that are adoptively transferred. In this study, the in vivo dose of 1 million macrophages per scaffold was selected through pilot studies to yield sufficient cell recovery for analysis by flow cytometry after 1 week of implantation, but a lower dose may be sufficient to influence muscle regeneration, while higher doses may lead to fibrosis or excessive type 2 inflammation.

The cell therapy platform demonstrated here can be expanded outside of VML and even beyond regenerative medicine. Macrophages are found across many tissue types and organs and play important roles in the immune response to various pathologies, including cancer, atherosclerosis, and fibrosis. The modularity of the proposed cell therapy stems from the ability to replace the mRNA being delivered to macrophages, or even deliver a combination of mRNAs. This strategy could therefore enable adoptively transferred macrophages to maintain virtually any desired phenotype, regardless of their microenvironment. Future studies should explore the use of this macrophage cell therapy in a variety of contexts where control over macrophage behavior is desired.

## 4. Methods

### 4.1. Human cell culture

Monocytes were isolated from peripheral blood using Percoll/Ficoll density gradient and cultured on non-tissue culture-treated plates in RPMI-1640 supplemented with 10 % human serum and 1 % penicillin/streptomycin. Media was additionally supplemented with 20 ng/mL of human macrophage colony-stimulating factor (Peprotech) to induce differentiation into macrophages. After 5 days, macrophages were maintained in an unactivated state or activated with 100 ng/mL each of interferon-gamma (Peprotech) and lipopolysaccharide (Sigma Aldrich) for 24 h.

### 4.2. Murine cell culture

Bone marrow was extracted from the femurs and tibias of wild-type C57Bl/6 J mice or transgenic GFP mice (Jackson Labs) and cultured on non-tissue culture-treated plates in RPMI-1640 supplemented with 10 % fetal bovine serum and 1 % penicillin/streptomycin. Media was additionally supplemented with 25 ng/mL of murine macrophage colony-stimulating factor (Peprotech) to induce differentiation into macrophages.

### 4.3. LNP fabrication and treatment

LNPs were formulated via microfluidic mixing as previously described [60]. Briefly, lipid components were combined in an ethanol phase and mRNA encoding luciferase or human or murine IL-4 was dissolved in citrate buffer (pH = 3) to form an aqueous phase. Syringe pumps were used to combine ethanol and aqueous phases via chaotic mixing at a 1:3 volumetric ratio. LNPs were then dialyzed against 1X PBS for 2 h and stored at 4 °C for later use.

Human (500ng/50k cells) or murine (25–100ng/50k cells) macrophages were incubated with LNPs for 12 h to facilitate phagocytosis. Human pro-inflammatory macrophages were maintained in IFNg/LPS-supplemented media after LNP treatment to model an inflammatory environment.

### 4.4. NanoString gene expression assay

24 h following the end of the 12-h LNP treatment period, human macrophages were lysed, RNA was extracted using the RNAqueous-Micro Total RNA Isolation Kit (ThermoFisher), and RNA concentration was measured using the Tecan Infinite M200 microplate reader. Gene expression was measured with custom-designed gene panels (Supplementary Table 1), using 100 ng of RNA per sample, according to nanoString’s protocol. Quality control analysis and raw data were normalized to in-house positive controls using nSolver 4.0 software (nanoString Technologies) as recommended by the manufacturer. Data was normalized to the total gene counts prior to analysis to account for variance in RNA quality.

### 4.5. Enzyme-linked immunosorbent assay

Cell-conditioned media was collected from 1 to 7 days after treatment of human macrophages with LNPs. For murine macrophages, media was collected immediately after treatment and daily for the following 6 days. Media was frozen at −80 °C until ELISAs were run. ELISAs were conducted using kits from Peprotech. Absorbance was measured on the Tecan Infinite M200 microplate reader.

### 4.6. Viability assay

Macrophages were removed from plates with cell scrapers, then 10uL of cell suspension was mixed 1:1 with Trypan Blue. Cells were counted with the Cell Countess (ThermoFisher) immediately following the addition of Trypan Blue.

### 4.7. Cell therapy treatment of volumetric muscle loss (VML)

4 mm biopsy punches were used to cut scaffolds from 2 mm thick porous gelatin sponge (Surgifoam) sheets (Ethicon). Scaffolds were hydrated in sterile PBS for at least 1 h prior to cell seeding.

Immediately following 12 h of treatment with LNPs, murine bone-marrow-derived unactivated macrophages were removed from plates with cell scrapers and centrifuged at 300×*g* for 5 min. Cells were resuspended in media at a volume of 1 million cells/10uL. 10uL of cell suspension was pipetted on top of prepared gelatin scaffolds, then incubated at 37 °C for 2 h to facilitate cell attachment.

All animal experiments were approved by the Drexel University Institutional Animal Care and Use Committee. Wild-type C57Bl/6 J mice, both male and female, were purchased at the age of 7–11 weeks, then acclimated for one week prior to surgery. Mice were anesthetized with isoflurane prior to and throughout surgery. Hair was removed from the left lower limb, which was then sterilized with iodine and isopropyl alcohol. A small incision was made to expose the quadriceps muscle, then a sterile 4 mm biopsy punch was used to create a critical sized defect in the muscle. Cell-seeded or unseeded scaffolds were immediately placed in the defect when applicable and skin was sutured closed.

### 4.8. Flow cytometry

7 days post-surgery, mice were euthanized and the entire injured quadriceps, still containing the scaffolds, and proximal inguinal lymph nodes were resected and placed in RPMI-1640. Muscle samples were minced with sharp scissors, then RPMI supplemented with collagenase type II (Worthington) was added for a final collagenase concentration of 3 mg/mL. Lymph nodes were broken up by crushing with a syringe plunger on scratched plates containing RPMI and collagenase type IV. Samples were placed in a 37 °C shaker incubator for 30 (lymph nodes) or 45 (muscle) minutes, then filtered through a 40um cell strainer and centrifuged at 600×*g* for 7 min. Cell pellet was resuspended in PBS and transferred to a 96-well U-bottom plate for staining.

Samples were stained with Live/Dead Fixable Aqua (Invitrogen) for 10 min, then incubated in Fc block for 10 min. Samples were next incubated in surface stain for 15 min, fixed and permeabilized overnight using the Foxp3/Transcription Buffer Staining Kit (Tonbo), then incubated in intracellular stain for 45 min. Fluorescence minus one (FMO) controls were made for each panel to assist with gating (Supplementary Figs. 17–19). Flow cytometry was conducted on the LSRFortessa (mouse in vitro and in vivo macrophage phenotyping) in the Sidney Kimmel Cancer Center at Thomas Jefferson University, or the BD FACSymphony A1 (in vivo leukocyte classification and T cell phenotyping) at the Cell Imaging Center at Drexel University. BD FACSDiva software was used to collect and FlowJo 10 software was used to analyze flow cytometry data.

### 4.9. Histology

28 days post-treatment, mice were euthanized, and the injured leg was removed and placed in 10 % neutral-buffered formalin for 24 h at room temperature. After fixation, the quadriceps was resected, trimmed, and stored in 70 % ethanol at 4 °C. Samples were embedded in paraffin wax, then 4-μm sections were made at 4 locations along the muscle. Samples were stained for Masson’s Trichrome, H&E, and single stains of laminin, CD31, F4/80, and GFP. Slides were imaged at 20X (Masson’s Trichrome, H&E) or 40X (IHC) magnification on the Aperio CS2 Scanscope (Leica), and images were processed in Aperio ImageScope and analyzed in ImageJ.

To measure scaffold area, the scale was set according to the scale bar and the area of the dark blue (collagen-positive) section of each Masson’s Trichrome-stained image was selected and measured. To narrow the scope of analysis to regenerating tissue, a region of interest (ROI) starting from the scaffold/muscle interface and continuing 200 μm into the muscle was isolated and analyzed. This ROI was used for the analysis of blood vessel, centrally nucleated (i.e. actively regenerating), and non-centrally nucleated muscle fibers counts as well as muscle fiber cross-sectional area (CSA). Blood vessels were manually counted as CD31^+^ lumens (i.e. closed circular cross-section). Laminin-stained slides exhibited clearly outlined muscle fibers and nuclei (Supplementary Fig. 18). Centrally nucleated and non-centrally nucleated muscle fibers were manually counted. Muscle fiber outlines were manually traced on the laminin-stained images within the ROI. CSA was calculated for each fiber using the traced images and custom ImageJ macro using the function “Analyze Particles.” CSA area was limited between 50 and 5000 μm^2^.

### 4.10. Statistics

Statistical analysis was conducted using Prism 10 software (Graph-Pad). All data is reported as mean ± standard deviation. RStudio was used to create heatmaps of genes with the lowest p-values. To account for false discovery rate from multiple testing, a Holm-Sidak-adjusted p-value of p < 0.05 was used to identify DEGs. In vitro human experiments used macrophages derived from n = 3 human donors. For in vitro mouse-derived macrophage experiments, a minimum of n = 3 experimental replicates were used per condition. Pair-wise comparisons were performed using Student’s t-tests. Multiple comparisons were determined using one-way ANOVA with Tukey’s post-hoc. For in vivo experiments, a minimum of n = 6 mice were used per condition. For fold-change results, statistical tests were conducted on log-transformed data. For fiber CSA distribution analysis, pairwise Kolmogorov–Smirnov distribution tests were performed between each condition (n = 9).

## Supplementary Material

Supplementary Material

## Data Availability

Data will be made available on request.

## Appendix A. Supplementary data

Supplementary data to this article can be found online at https://doi.org/10.1016/j.biomaterials.2025.123869.
