# Supplementary material for: Macrophage cell therapy enabled by interleukin-4 mRNA-loaded lipid nanoparticles to sustain a pro-reparative phenotype in inflammatory injuries
Source: Biomaterials. Author manuscript; Available in PMC 2026 Apr 27. (PMC13112448; doi:10.1016/j.biomaterials.2025.123869)
Supplement: Supplementary Material [file NIHMS2163147-supplement-Supplementary_Material.pdf]

Macrophage cell therapy enabled by mRNA-loaded lipid nanoparticles to sustain a pro-reparative phenotype in inflammatory injuries

Erin M. O'Brien, Tina Tylek, Alvin J. Mukalel, Ricardo C. Whitaker, Hannah Geisler, Benjamin I. Binder-Markey, Drew Weissman, Michael J. Mitchell, Kara L. Spiller

a

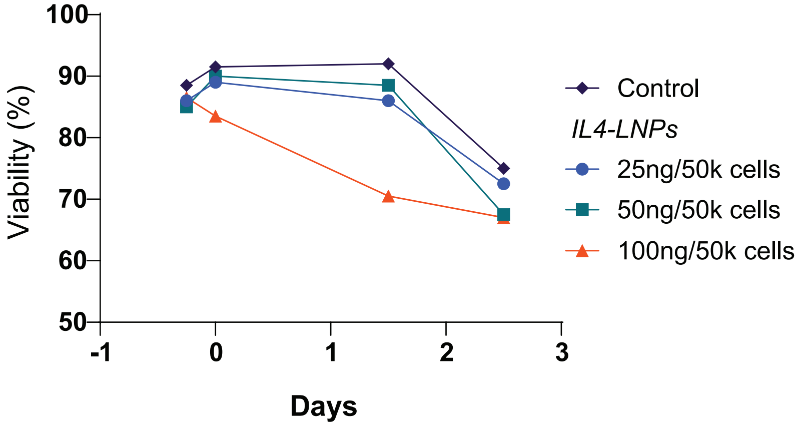

b

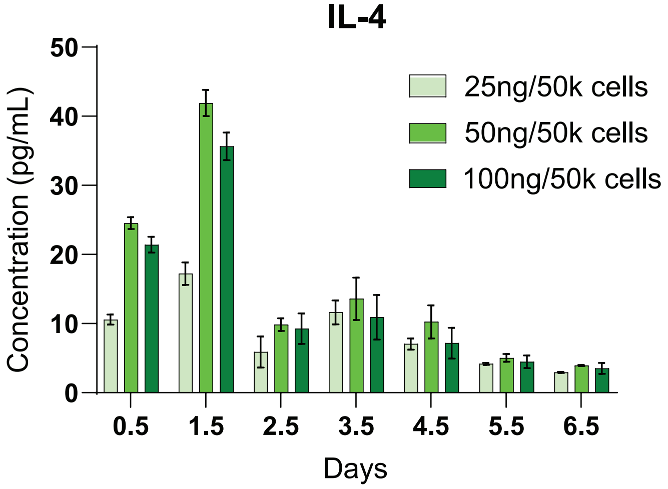

Supplementary Figure 1. Effects of IL4-LNPs on primary murine macrophages in vitro. (a) Viability over time before and after treatment with varying doses of LNPs. (b) Macrophage secretion of IL-4 during incubation with varying doses of IL4-LNPs, and 6 days following the end of incubation period.

Table 1: Custom Nanostring panel

Probe Name	NS Probe ID	Class Name	Analyte Type
ABCG2	NM_004827.2:2025	Endogenous	mRNA
ACOD1	NM_001258406.1:1334	Endogenous	mRNA
ACTA2	NM_001613.1:645	Endogenous	mRNA
AGGF1	NM_018046.3:35	Endogenous	mRNA
ALDOA	NM_184041.2:1455	Endogenous	mRNA
ALOX15	NM_001140.3:1910	Endogenous	mRNA
ANG	NM_001145.4:949	Endogenous	mRNA
APOBEC3A	NM_145699.3:321	Endogenous	mRNA
ATG5	NM_004849.2:1104	Endogenous	mRNA
ABCA9	NM_080283.3:2614	Endogenous	mRNA
APOE	NM_000041.2:96	Endogenous	mRNA
AXL	NM_021913.2:2190	Endogenous	mRNA
BAX	NM_138761.3:342	Endogenous	mRNA
BGN	NM_001711.3:1935	Endogenous	mRNA
BTG1	NM_001731.2:775	Endogenous	mRNA
BCL2	NM_000657.2:5	Endogenous	mRNA
C3AR1	NM_004054.2:415	Endogenous	mRNA
CABLES1	NM_001100619.2:2560	Endogenous	mRNA
CACNA1G	NM_198397.1:1380	Endogenous	mRNA
CACNB4	NM_000726.3:504	Endogenous	mRNA
SHPK	NM_013276.2:1590	Endogenous	mRNA
CCL1	NM_002981.1:157	Endogenous	mRNA
CCL15	NM_032965.4:869	Endogenous	mRNA
CCL17	NM_002987.2:229	Endogenous	mRNA
CCL18	NM_002988.2:585	Endogenous	mRNA
CCL19	NM_006274.2:401	Endogenous	mRNA
CCL2	NM_002982.3:123	Endogenous	mRNA
CCL20	NM_004591.1:35	Endogenous	mRNA
CCL22	NM_002990.3:797	Endogenous	mRNA
CCL24	NM_002991.2:18	Endogenous	mRNA
CCL26	NM_006072.4:184	Endogenous	mRNA
CCL4	NM_002984.2:201	Endogenous	mRNA
CCL5	NM_002985.2:280	Endogenous	mRNA
CCL8	NM_005623.2:689	Endogenous	mRNA

CCN2	NM_001901.2:1100	Endogenous	mRNA
CCR10	NM_016602.1:1115	Endogenous	mRNA
CCR2	NM_001123041.2:743	Endogenous	mRNA
CCR7	NM_001838.2:1610	Endogenous	mRNA
CCR8	NM_005201.2:245	Endogenous	mRNA
ITGAM	NM_000632.3:515	Endogenous	mRNA
SLAMF1	NM_003037.2:580	Endogenous	mRNA
FCGR3A	NM_000569.6:1644	Endogenous	mRNA
CD163	NM_004244.4:1630	Endogenous	mRNA
ALCAM	NM_001627.3:789	Endogenous	mRNA
CD1C	NM_001765.2:750	Endogenous	mRNA
CD200R1	NM_138806.3:142	Endogenous	mRNA
PDCD1LG2	NM_025239.3:235	Endogenous	mRNA
CD274	NM_014143.3:49	Endogenous	mRNA
CD36	NM_000072.3:707	Endogenous	mRNA
CD38	NM_001775.2:1035	Endogenous	mRNA
CD68	NM_001251.2:1140	Endogenous	mRNA
CD80	NM_005191.3:1288	Endogenous	mRNA
CD93	NM_012072.3:4270	Endogenous	mRNA
CH25H	NM_003956.3:532	Endogenous	mRNA
CLEC10A	NM_182906.2:430	Endogenous	mRNA
COL11A1	NM_001190709.1:2490	Endogenous	mRNA
COL1A1	NM_000088.3:5210	Endogenous	mRNA
COL3A1	NM_000090.3:180	Endogenous	mRNA
COL5A1	NM_000093.3:872	Endogenous	mRNA
CREB1	NM_004379.3:618	Endogenous	mRNA
CTNNB1	NM_001098210.1:1815	Endogenous	mRNA
CX3CR1	NM_001337.3:1040	Endogenous	mRNA
CXCL10	NM_001565.2:461	Endogenous	mRNA
CXCL11	NM_005409.4:282	Endogenous	mRNA
CXCL12	NM_199168.3:69	Endogenous	mRNA
CXCL2	NM_002089.3:854	Endogenous	mRNA
CXCL3	NM_002090.2:540	Endogenous	mRNA
CXCL9	NM_002416.1:1975	Endogenous	mRNA
CXCR2	NM_001557.2:2055	Endogenous	mRNA
CXCR4	NM_003467.2:1335	Endogenous	mRNA
CCN1	NM_001554.3:1390	Endogenous	mRNA

PTGS2	NM_000963.1:495	Endogenous	mRNA
DACT1	NM_001079520.1:3350	Endogenous	mRNA
DCN	NM_001920.3:420	Endogenous	mRNA
DNASE1L3	NM_001256560.1:478	Endogenous	mRNA
EGFL7	NM_016215.3:1252	Endogenous	mRNA
EGR2	NM_000399.3:1891	Endogenous	mRNA
ENO1	NM_001428.2:1689	Endogenous	mRNA
EPAS1	NM_001430.3:4246	Endogenous	mRNA
ERG	NM_182918.3:697	Endogenous	mRNA
ETS1	NM_005238.3:1305	Endogenous	mRNA
FBP1	NM_000507.3:590	Endogenous	mRNA
FGF2	NM_002006.4:620	Endogenous	mRNA
FLT1	NM_002019.4:530	Endogenous	mRNA
FN1	NM_212482.1:1776	Endogenous	mRNA
FOXO1	NM_002015.3:1526	Endogenous	mRNA
FOXO3	NM_001455.2:1860	Endogenous	mRNA
FOXO4	NM_001170931.1:1121	Endogenous	mRNA
FST	NM_006350.2:575	Endogenous	mRNA
FSTL1	NM_007085.4:1610	Endogenous	mRNA
FYN	NM_002037.3:765	Endogenous	mRNA
LGALS3	NM_001177388.1:495	Endogenous	mRNA
GATA3	NM_001002295.1:1691	Endogenous	mRNA
GLUL	NM_001033044.2:2645	Endogenous	mRNA
HIF1A	NM_001530.2:1985	Endogenous	mRNA
HLA-DRA	NM_019111.3:335	Endogenous	mRNA
HSPG2	NM_005529.5:2715	Endogenous	mRNA
IDO1	NM_002164.5:369	Endogenous	mRNA
IDO2	NM_194294.2:1575	Endogenous	mRNA
IGF1	NM_000618.3:491	Endogenous	mRNA
IL13	NM_002188.2:516	Endogenous	mRNA
IL10	NM_000572.2:622	Endogenous	mRNA
IL10RB	NM_000628.3:1760	Endogenous	mRNA
IL12A	NM_000882.2:775	Endogenous	mRNA
IL12B	NM_002187.2:1435	Endogenous	mRNA
IL13RA1	NM_001560.2:1230	Endogenous	mRNA
IL13RA2	NM_000640.2:400	Endogenous	mRNA

IL17A	NM_002190.2:240	Endogenous	mRNA
IL1B	NM_000576.2:840	Endogenous	mRNA
IL1R1	NM_001320984.1:281	Endogenous	mRNA
IL4	NM_000589.2:625	Endogenous	mRNA
IL4R	NM_000418.3:1510	Endogenous	mRNA
IL6	NM_000600.3:364	Endogenous	mRNA
CXCL8	NM_000584.2:25	Endogenous	mRNA
IRF4	NM_002460.1:325	Endogenous	mRNA
IRF5	NM_002200.3:1845	Endogenous	mRNA
ITGB1BP1	NM_004763.3:1690	Endogenous	mRNA
JAK2	NM_004972.3:1464	Endogenous	mRNA
JUN	NM_002228.3:140	Endogenous	mRNA
JAG1	NM_000214.2:915	Endogenous	mRNA
JAK1	NM_002227.1:285	Endogenous	mRNA
JAK3	NM_000215.2:1715	Endogenous	mRNA
KLF4	NM_004235.4:1980	Endogenous	mRNA
TNFSF14	NM_003807.3:350	Endogenous	mRNA
LIN7A	NM_004664.2:1027	Endogenous	mRNA
LUM	NM_002345.3:1285	Endogenous	mRNA
NRIH3	NM_005693.2:1575	Endogenous	mRNA
NRIH2	NM_007121.4:30	Endogenous	mRNA
LRG1	NM_052972.3:656	Endogenous	mRNA
MARCO	NM_006770.3:61	Endogenous	mRNA
MCTP2	NM_001159644.1:576	Endogenous	mRNA
MERTK	NM_006343.2:665	Endogenous	mRNA
MEST	NM_177525.1:645	Endogenous	mRNA
MMP13	NM_002427.2:951	Endogenous	mRNA
MMP2	NM_004530.2:2360	Endogenous	mRNA
MMP7	NM_002423.3:311	Endogenous	mRNA
MMP8	NM_002424.2:2590	Endogenous	mRNA
MMP9	NM_004994.2:1530	Endogenous	mRNA
MRC1	NM_002438.2:525	Endogenous	mRNA
MS4A6E	NM_139249.2:221	Endogenous	mRNA
NAA15	NM_057175.3:4145	Endogenous	mRNA
NFKB1	NM_003998.2:1675	Endogenous	mRNA
NNMT	NM_006169.2:1069	Endogenous	mRNA
NOD1	NM_006092.2:178	Endogenous	mRNA
NOD2	NM_001293557.1:1120	Endogenous	mRNA

OLR1	NM_002543.3:295	Endogenous	mRNA
PDGFA	NM_002607.5:2460	Endogenous	mRNA
PDGFB	NM_033016.2:1480	Endogenous	mRNA
PDGFC	NM_016205.2:2596	Endogenous	mRNA
PDGFRA	NM_006206.3:1925	Endogenous	mRNA
PDGFRB	NM_002609.3:265	Endogenous	mRNA
PECAM1	NM_000442.3:1365	Endogenous	mRNA
PFKFB1	NM_002625.2:564	Endogenous	mRNA
PFKFB3	NM_004566.3:965	Endogenous	mRNA
PKM	NM_182471.1:2105	Endogenous	mRNA
PLOD2	NM_182943.2:2590	Endogenous	mRNA
PPARD	NM_006238.4:895	Endogenous	mRNA
PPARG	NM_005037.5:345	Endogenous	mRNA
PTGES2	NM_025072.6:1712	Endogenous	mRNA
AGER	NM_001136.3:340	Endogenous	mRNA
RAMP1	NM_005855.2:200	Endogenous	mRNA
RIPK3	NM_006871.3:1540	Endogenous	mRNA
S100A8	NM_002964.3:115	Endogenous	mRNA
SERPINA1	NM_000295.4:760	Endogenous	mRNA
SH3PXD2B	NM_001017995.2:228	Endogenous	mRNA
SOCS1	NM_003745.1:1025	Endogenous	mRNA
SOCS3	NM_003955.3:1870	Endogenous	mRNA
SPHK1	NM_021972.2:895	Endogenous	mRNA
SPP1	NM_000582.2:760	Endogenous	mRNA
SREBF1	NM_001005291.1:1392	Endogenous	mRNA
STAT3	NM_003150.3:2060	Endogenous	mRNA
STAT6	NM_003153.3:2030	Endogenous	mRNA
STAT1	NM_007315.3:1795	Endogenous	mRNA
TGFB1	NM_000660.3:1260	Endogenous	mRNA
TGFB3	NM_003239.2:706	Endogenous	mRNA
TGM2	NM_004613.2:590	Endogenous	mRNA
TIE1	NM_005424.4:2476	Endogenous	mRNA
TIMP1	NM_003254.2:329	Endogenous	mRNA
TIMP2	NM_003255.4:1555	Endogenous	mRNA
TIMP3	NM_000362.4:1640	Endogenous	mRNA
TNFAIP8L2	NM_024575.3:709	Endogenous	mRNA
TLR2	NM_003264.3:2402	Endogenous	mRNA
TLR3	NM_003265.2:230	Endogenous	mRNA

TLR4	NM_138554.2:2570	Endogenous	mRNA
TLR7	NM_016562.3:4120	Endogenous	mRNA
TNF	NM_000594.2:1010	Endogenous	mRNA
TNFAIP6	NM_007115.2:250	Endogenous	mRNA
TNFRSF1B	NM_001066.2:835	Endogenous	mRNA
TNFRSF11A	NM_003839.3:226	Endogenous	mRNA
TNFRSF1A	NM_001065.2:515	Endogenous	mRNA
TRAF6	NM_145803.2:745	Endogenous	mRNA
TYK2	NM_003331.4:1980	Endogenous	mRNA
TERF1	NM_005410.2:200	Endogenous	mRNA
VCAN	NM_004385.3:9915	Endogenous	mRNA
VEGFA	NM_001025366.1:1325	Endogenous	mRNA
VEGFB	NM_003377.3:687	Endogenous	mRNA
VEGFC	NM_005429.2:565	Endogenous	mRNA
VIM	NM_003380.2:694	Endogenous	mRNA
WNT5A	NM_003392.3:475	Endogenous	mRNA
CD209	NM_001144899.1:950	Endogenous	mRNA
IRF1	NM_002198.2:15	Endogenous	mRNA
MORC4	NM_024657.3:1400	Endogenous	mRNA
ACTB	NM_001101.2:1010	Housekeeping	mRNA
CYC1	NM_001916.4:344	Housekeeping	mRNA
EIF2B2	NM_014239.2:625	Housekeeping	mRNA
GUSB	NM_000181.3:1899	Housekeeping	mRNA
HPRT1	NM_000194.1:240	Housekeeping	mRNA
TBP	NM_001172085.1:587	Housekeeping	mRNA
NEG_A	ERCC_00096.1:230	Negative	SYSTEM
NEG_B	ERCC_00041.1:440	Negative	SYSTEM
NEG_C	ERCC_00019.1:140	Negative	SYSTEM
NEG_D	ERCC_00076.1:355	Negative	SYSTEM

NEG_E	ERCC_00098.1:785	Negative	SYSTEM
NEG_F	ERCC_00126.1:220	Negative	SYSTEM
NEG_G	ERCC_00144.1:15	Negative	SYSTEM
NEG_H	ERCC_00154.1:115	Negative	SYSTEM
POS_A	ERCC_00117.1:385	Positive	SYSTEM
POS_B	ERCC_00112.1:695	Positive	SYSTEM
POS_C	ERCC_00002.1:850	Positive	SYSTEM
POS_D	ERCC_00092.1:540	Positive	SYSTEM
POS_E	ERCC_00035.1:485	Positive	SYSTEM
POS_F	ERCC_00034.1:195	Positive	SYSTEM

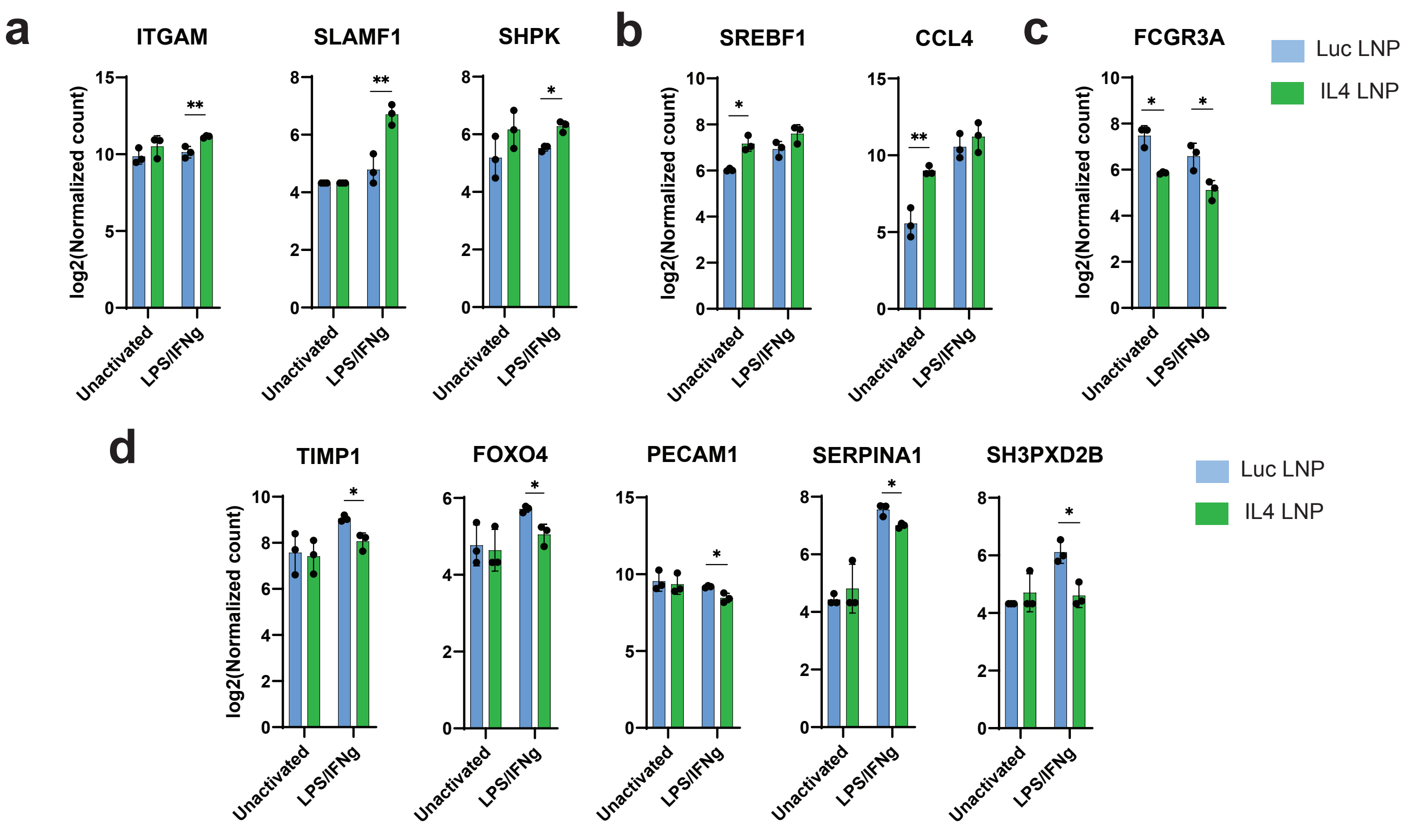

Supplementary Figure 2. Additional DEGs expressed by unactivated and/or pro-inflammatory macrophages 24 hours after treatment with IL4-LNPs, compared to Luc-LNPs. (a) Genes upregulated by pro-inflammatory but not unactivated macrophages. Data are represented as mean ± SD. Multiple t-tests with Holm-Sidak adjusted p-value, n=3 experimental replicates, *$p<0.05$, **$p<0.01$. (b) Genes upregulated by unactivated but not pro-inflammatory macrophages. (c) Genes downregulated by both unactivated and pro-inflammatory macrophages. (d) Genes downregulated by pro-inflammatory but not unactivated macrophages.

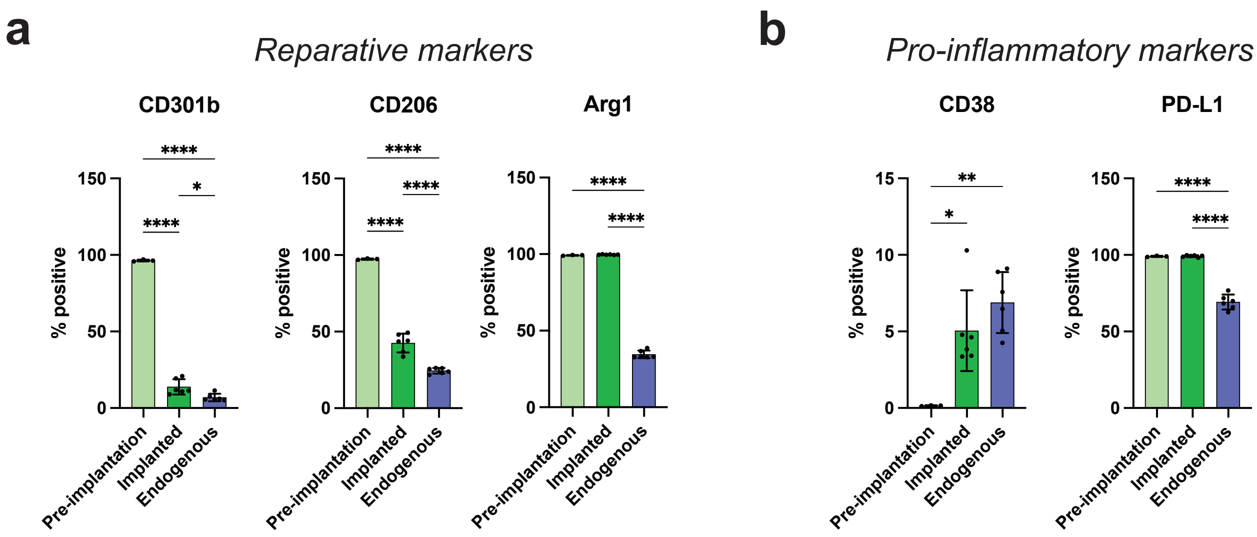

Supplementary Figure 3. Phenotype of adoptively transferred macrophages exogenously IL-4-polarized in a murine model of volumetric muscle loss. (a) Expression of reparative markers by IL-4-activated macrophages before and 3 days after implantation in a VML injury, compared to endogenous macrophages. Data are represented as mean ± SD. One-way ANOVA with Tukey's post-hoc, n=3-6 experimental replicates, *p<0.05, **p<0.01, ***p<0.0001. (b) Expression of pro-inflammatory markers.

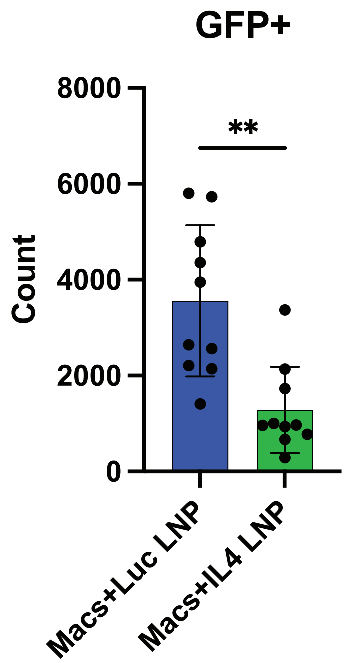

Supplementary Figure 4. Approximate GFP+ macrophage recovery one week following implantation in murine VML injury. Data are represented as mean ± SD. Student's t-test, n=10 mice, **$p<0.01$. Counts should not be considered absolute due to the loss of cells that occurs during tissue processing for flow cytometry.

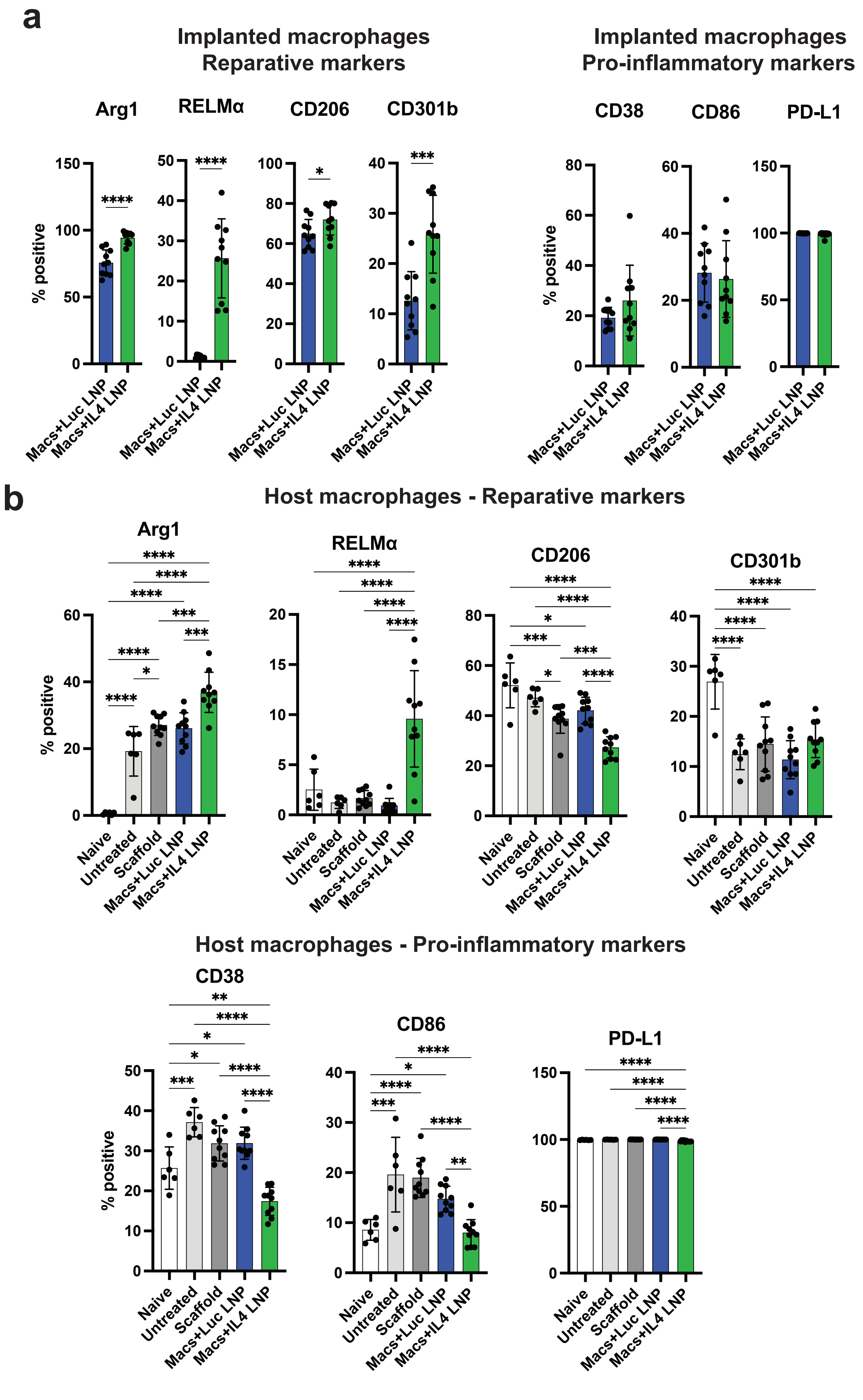

Supplementary Figure 5. Phenotype of adoptively transferred and endogenous macrophages in a murine model of volumetric muscle loss treated with IL4-LNP cell therapy, % positive. Expression of reparative and pro-inflammatory markers by (a) implanted and b) endogenous macrophages one week following implantation in VML model. Multiple t-tests with Holm-Sidak adjustment, n=3-5 mice, *padj<0.05.

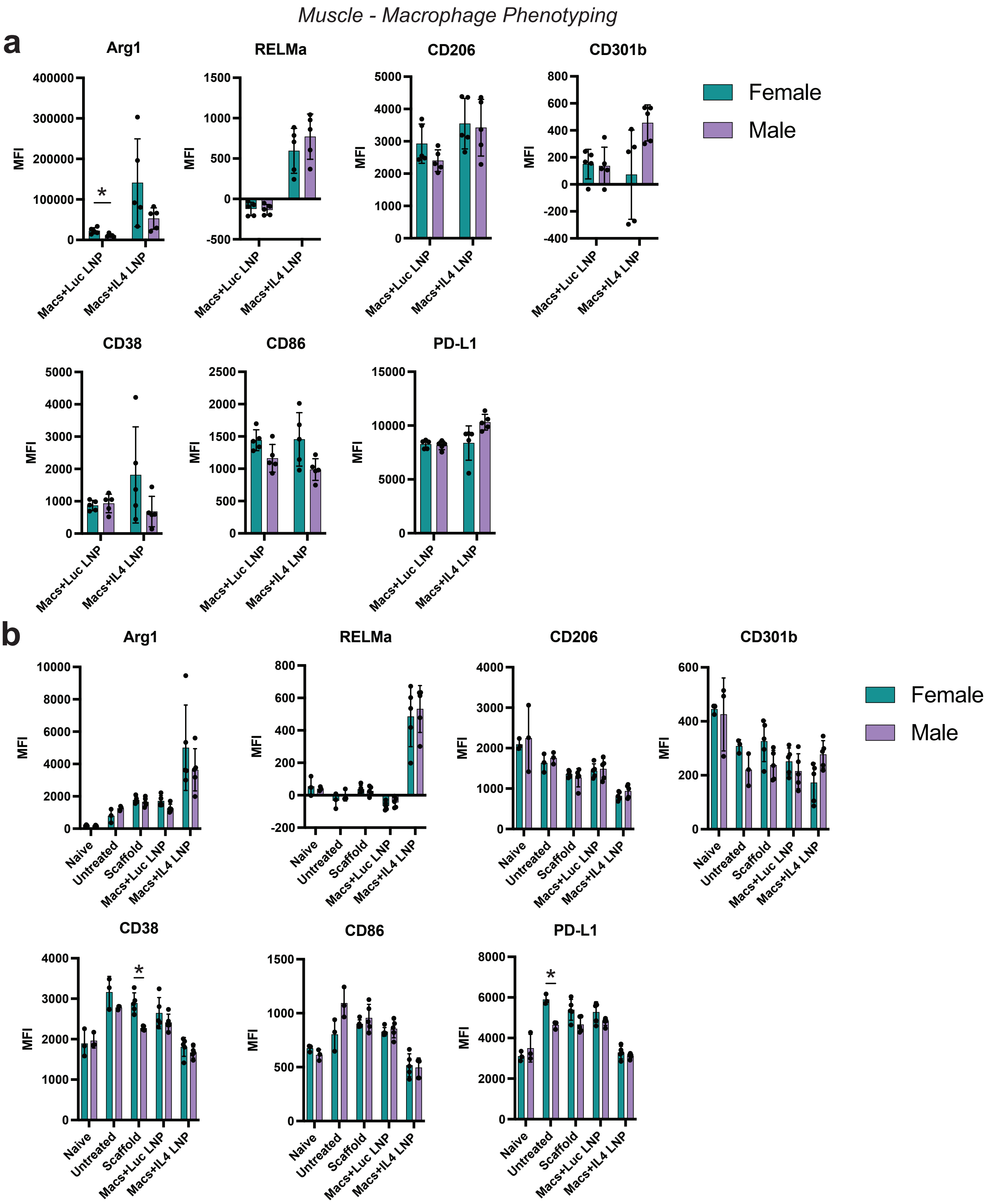

Supplementary Figure 6. Phenotype of adoptively transferred and endogenous macrophages in a murine model of volumetric muscle loss treated with IL4-LNP cell therapy, stratified by sex. Expression of reparative and pro-inflammatory markers by (a) implanted and b) endogenous macrophages one week following implantation in VML model. Multiple t-tests with Holm-Sidak adjustment, n=3-5 mice, *padj<0.05.

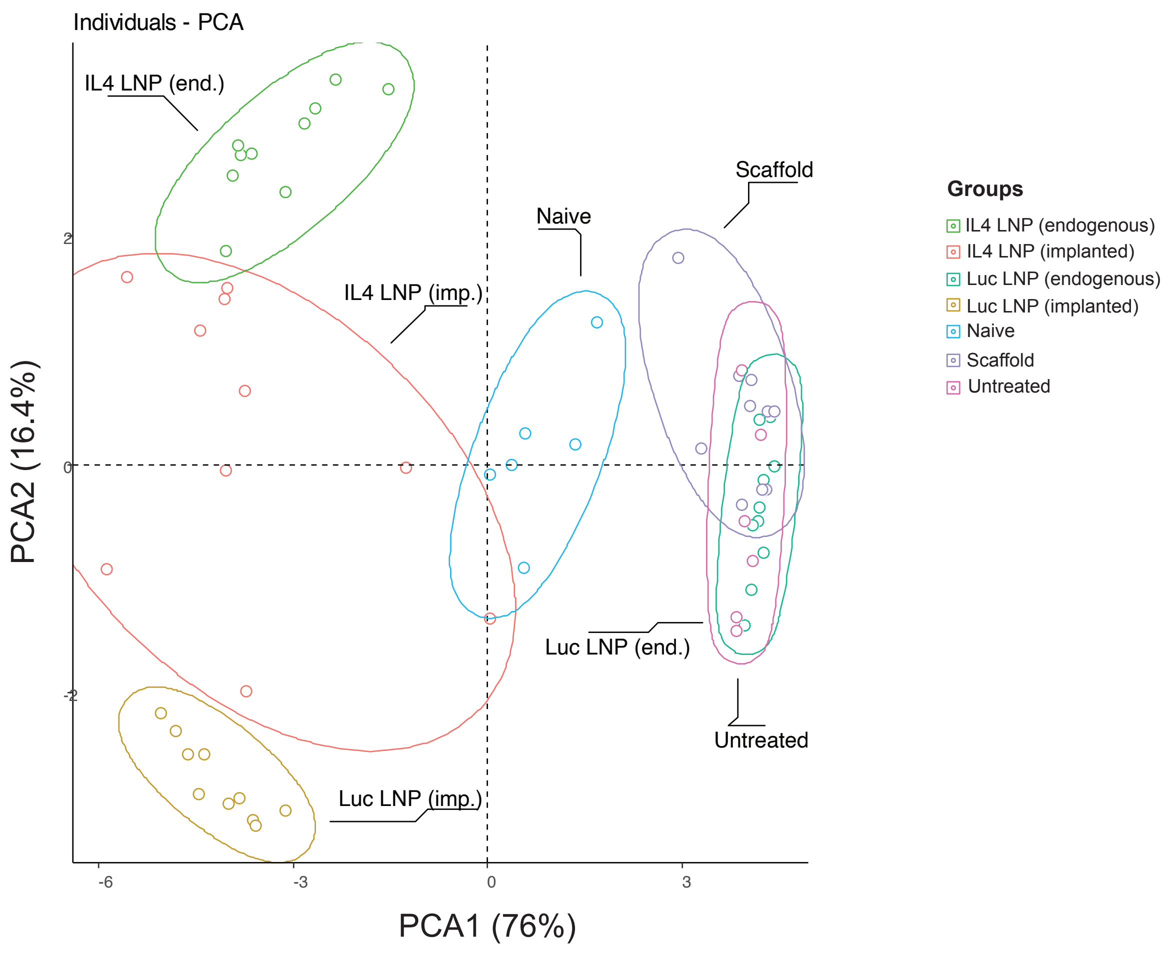

Supplementary Figure 7. Principal component analysis of pro-inflammatory and reparative marker expression by host (endogenous) and implanted macrophages one week following VML injury and treatment.

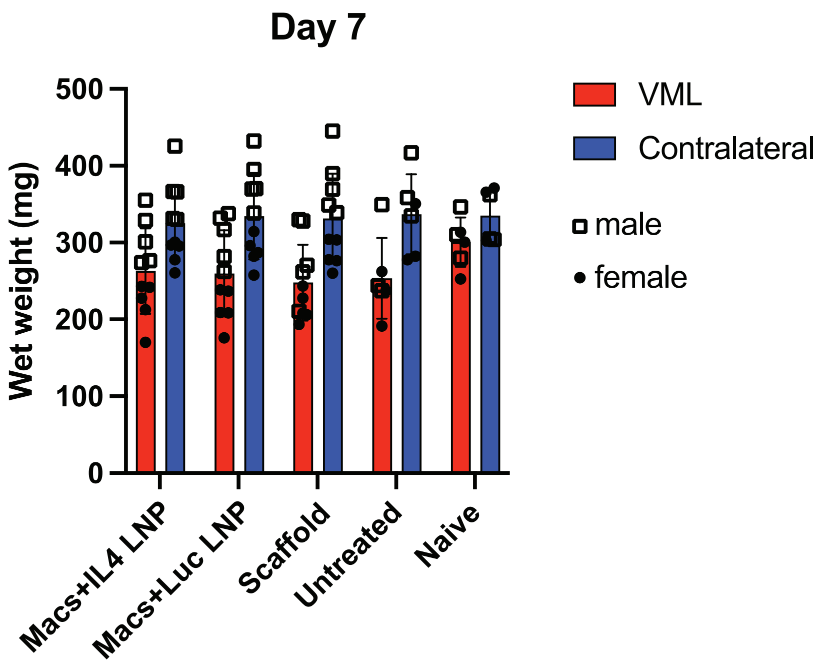

Supplementary Figure 8. Wet muscle weights of injured and contralateral muscles 7 days after VML injury. Data are represented as mean ± SD. n=6-10 biological replicates, *p<0.05, **p<0.01.

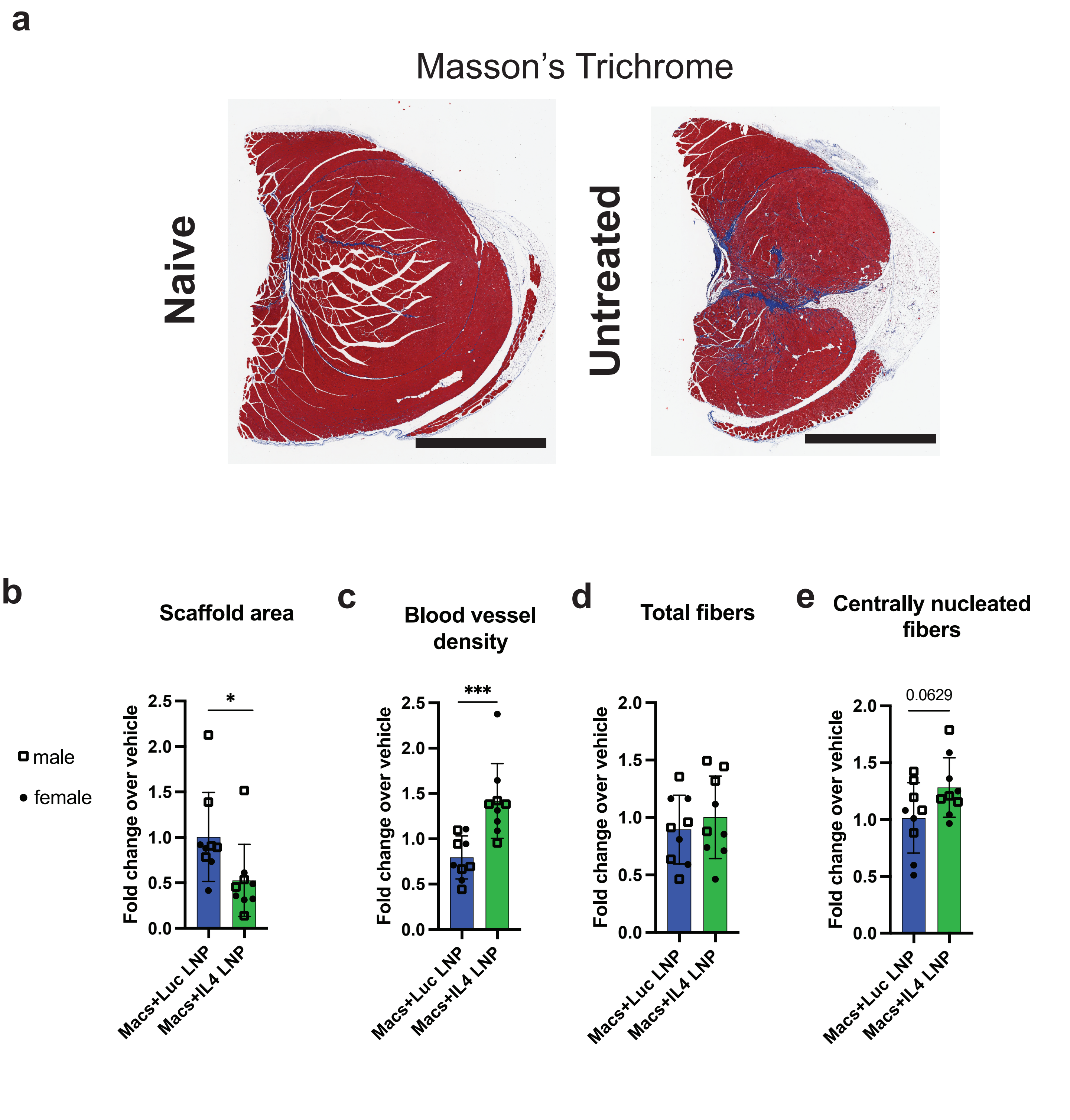

Supplementary Figure 9. Histology of muscle repair 28 days after VML injury.
(a) Representative images of naive (uninjured) and untreated muscle stained for Masson's Trichrome.
(b) Histological outcomes as fold-change over scaffold-only samples. Statistical testing was conducted on log-transformed data. Data are represented as mean ± SD. Student's t-test, n=9 mice, *p<0.05.

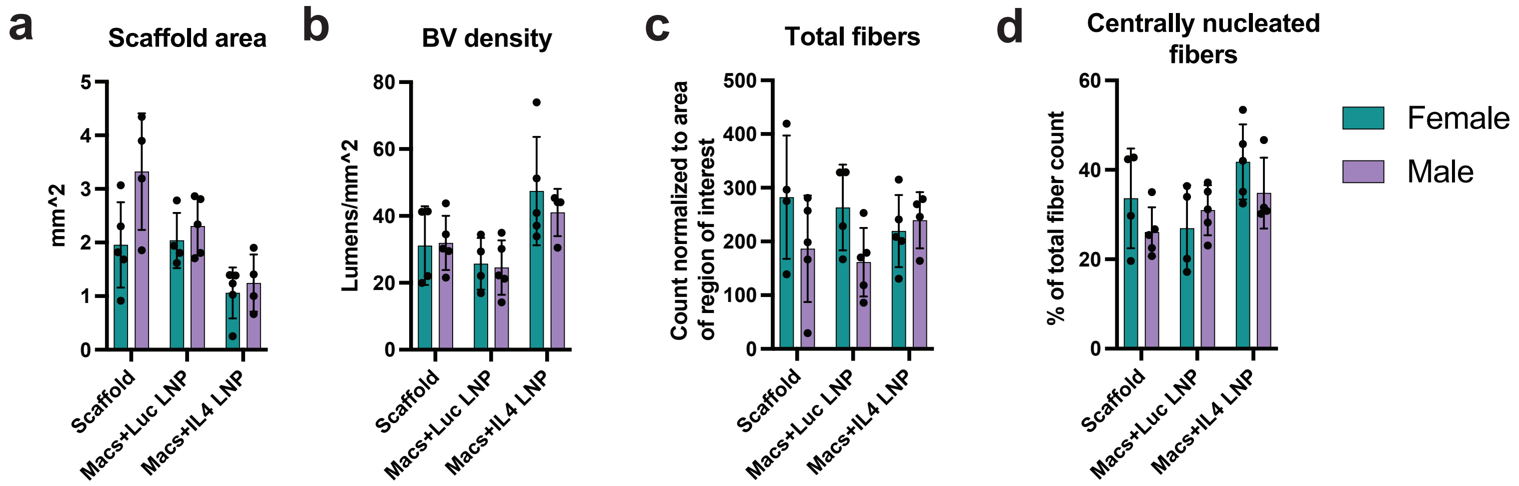

Supplementary Figure 10. Histological analysis of muscle repair, stratified by sex.
(a) Area of implant remaining at 28 days following VML injury and treatment. Data are represented as mean ± SD. Multiple t-tests with Solm-Hidak correction, n=4-5 mice, *padj<0.05.
(b) Density of CD31+ blood vessel lumens.
(c) Total muscle fiber count identified via laminin staining.
(d) Proportion of fibers that are centrally nucleated.

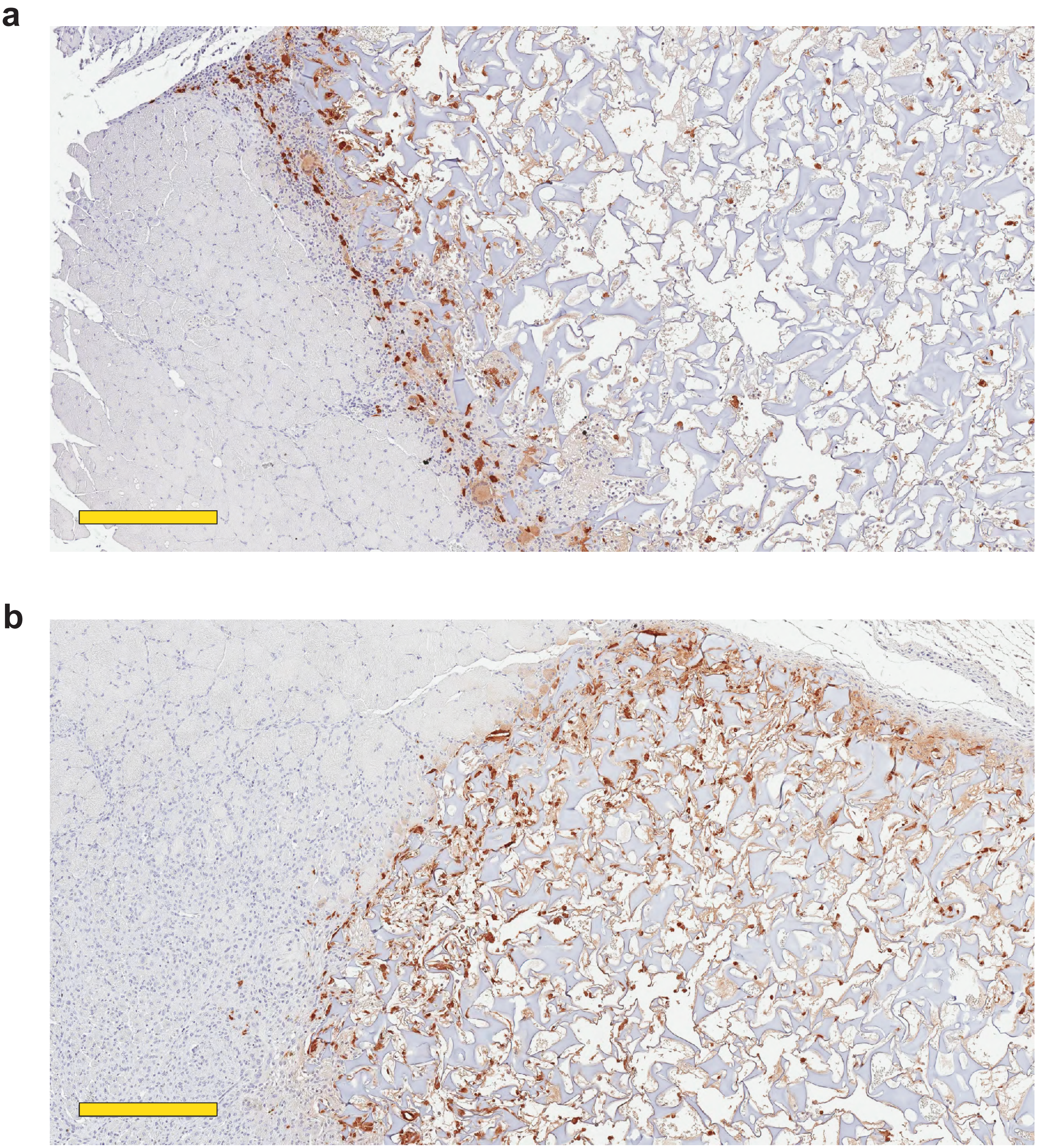

Supplementary Figure 11. Adoptively transferred GFP+ cells remain one week after implantation. (a) Representative image of histology section from IL4-LNP group stained for GFP (implanted cells). Scale bar=300 μm. (b) Representative image of histology section from Luc-LNP group stained for GFP.

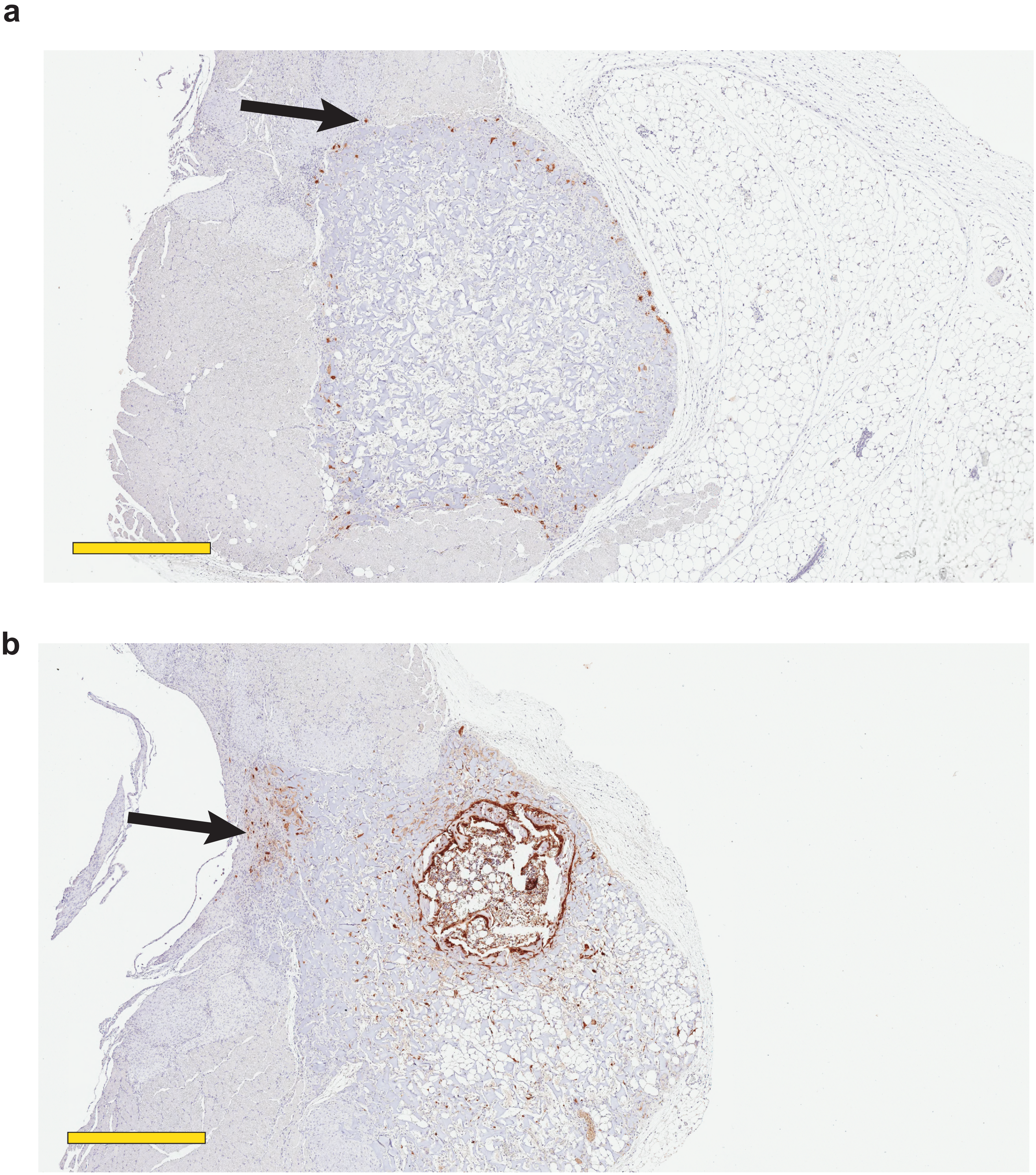

Supplementary Figure 12. Adoptively transferred GFP+ cell infiltration into muscle 28 days after implantation. (a) Representative image of histology section from IL4-LNP group stained for GFP. Scale bar=600 μm. Black arrows indicate areas outside of the scaffold where GFP+ cells have migrated. (b) Representative image of histology section from Luc-LNP group stained for GFP.

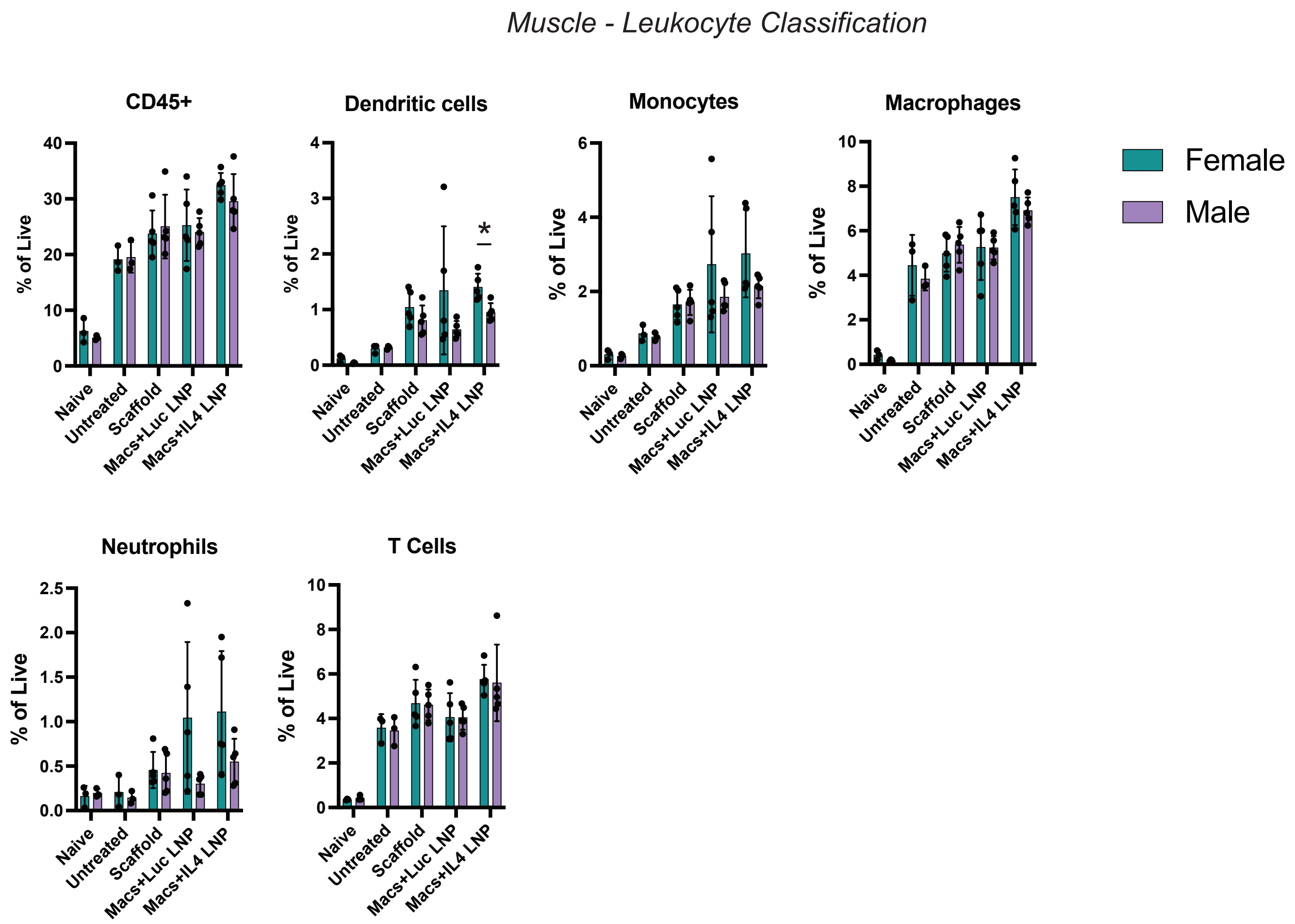

Supplementary Figure 13. Classification of muscle-infiltrating leukocytes one week following VML injury and treatment, stratified by sex. Multiple t-tests withHolm-Sidak adjustment, n=3-5 mice, *padj<0.05.

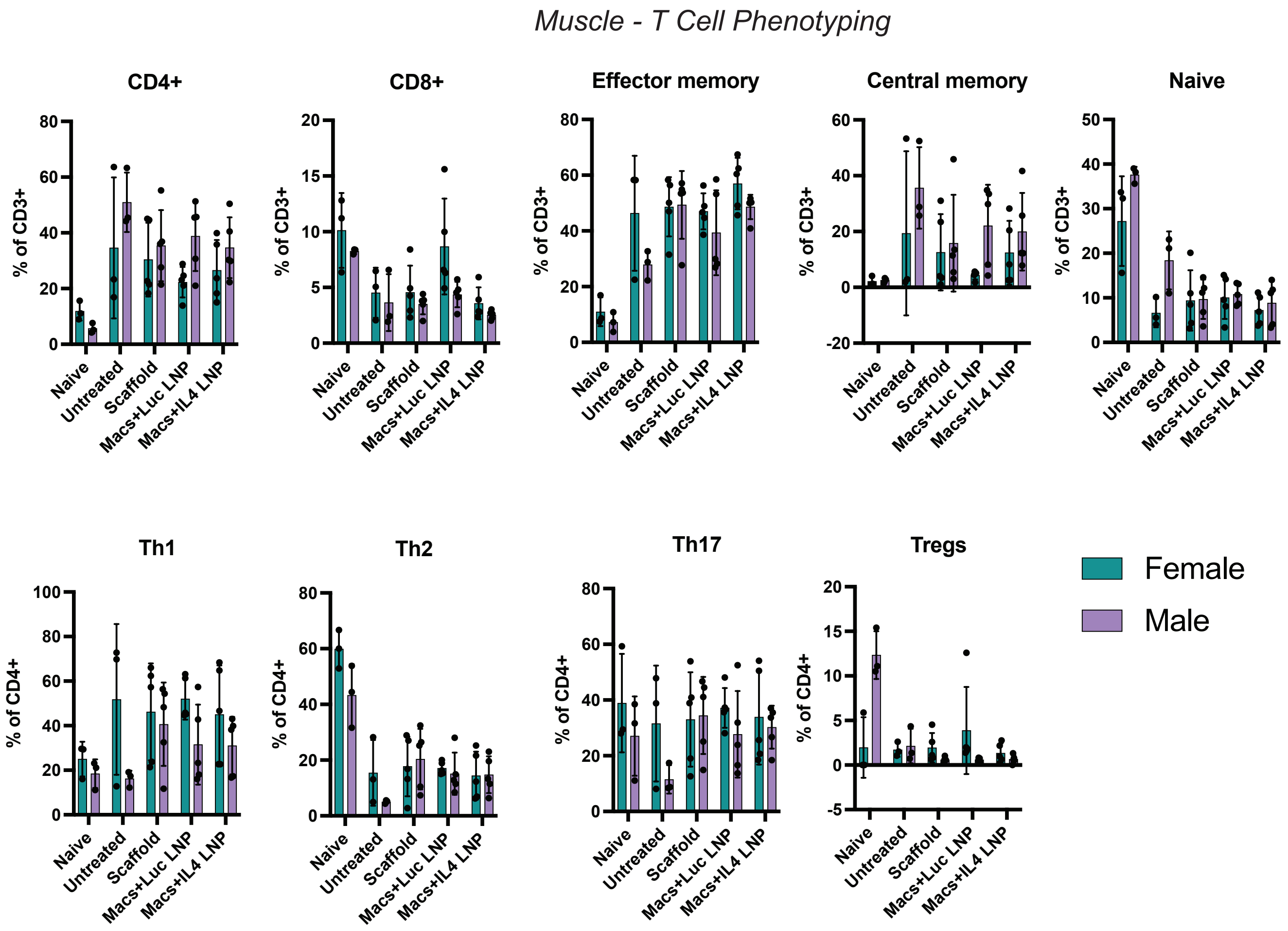

Supplementary Figure 14. Phenotyping of muscle-infiltrating T cells one week following VML injury and treatment, stratified by sex. Multiple t-tests with Holm-Sidak adjustment, n=3-5 mice, *padj<0.05.

Draining lymph node - T Cell Phenotyping

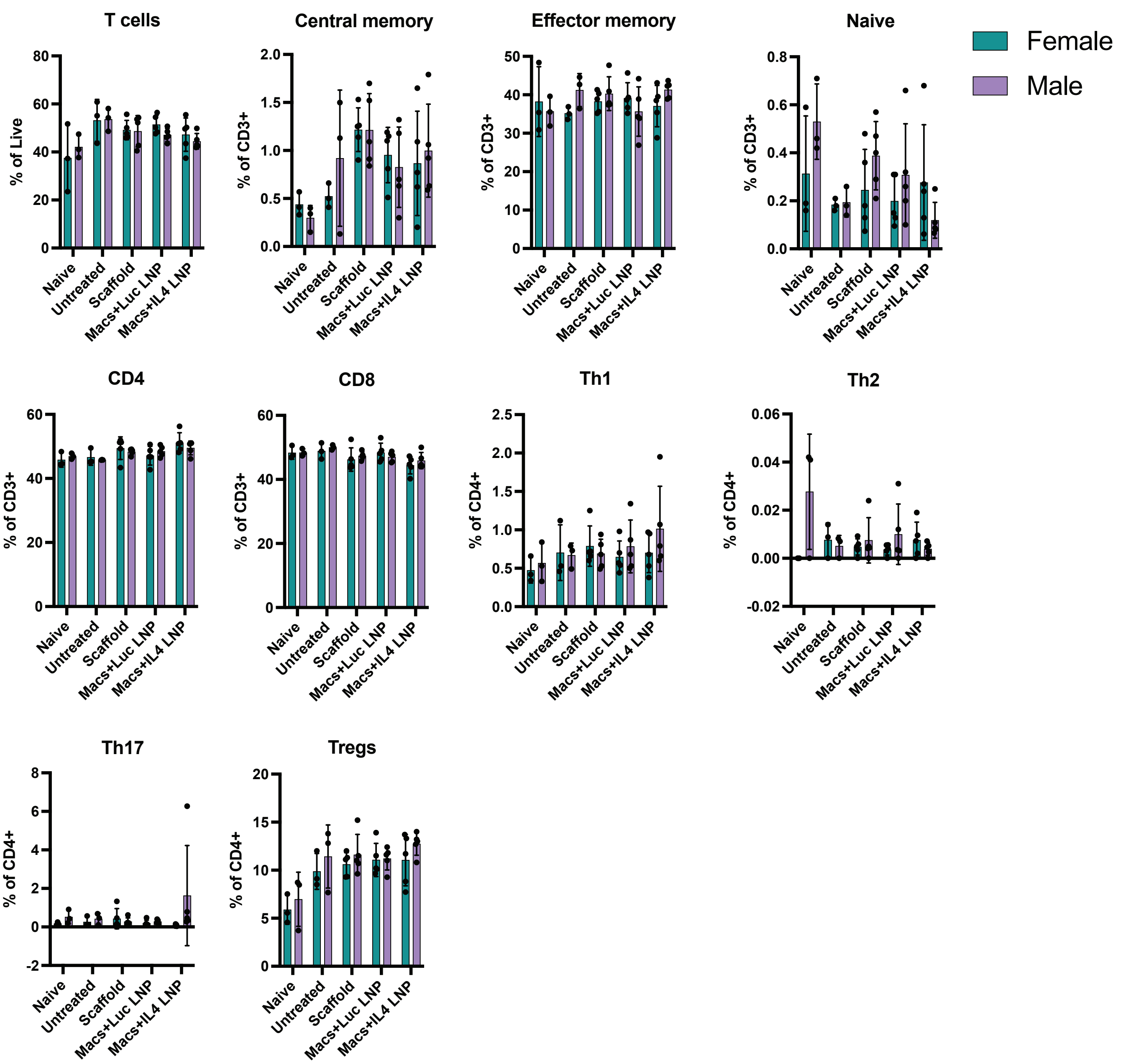

Supplementary Figure 15. Phenotyping of T cells in draining inguinal lymph nodes one week following VML injury and treatment, stratified by sex. Multiple t-tests with Holm-Sidak adjustment, n=3-5 mice, *padj<0.05.

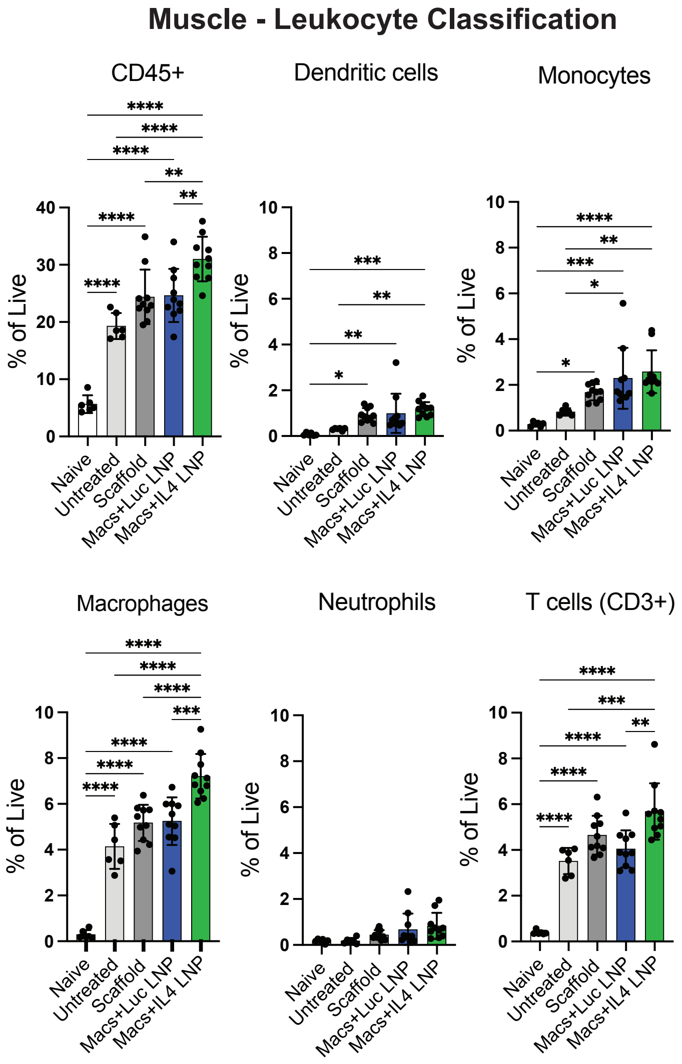

Supplementary Figure 16. Classification of muscle-infiltrating leukocytes one week following VML injury and treatment, with all significant differences shown. Data are represented as mean ± SD. One-way ANOVA with Tukey's post-hoc, n=6-10 mice, *p<0.05, **p<0.01, ***p<0.001, ****p<0.0001.

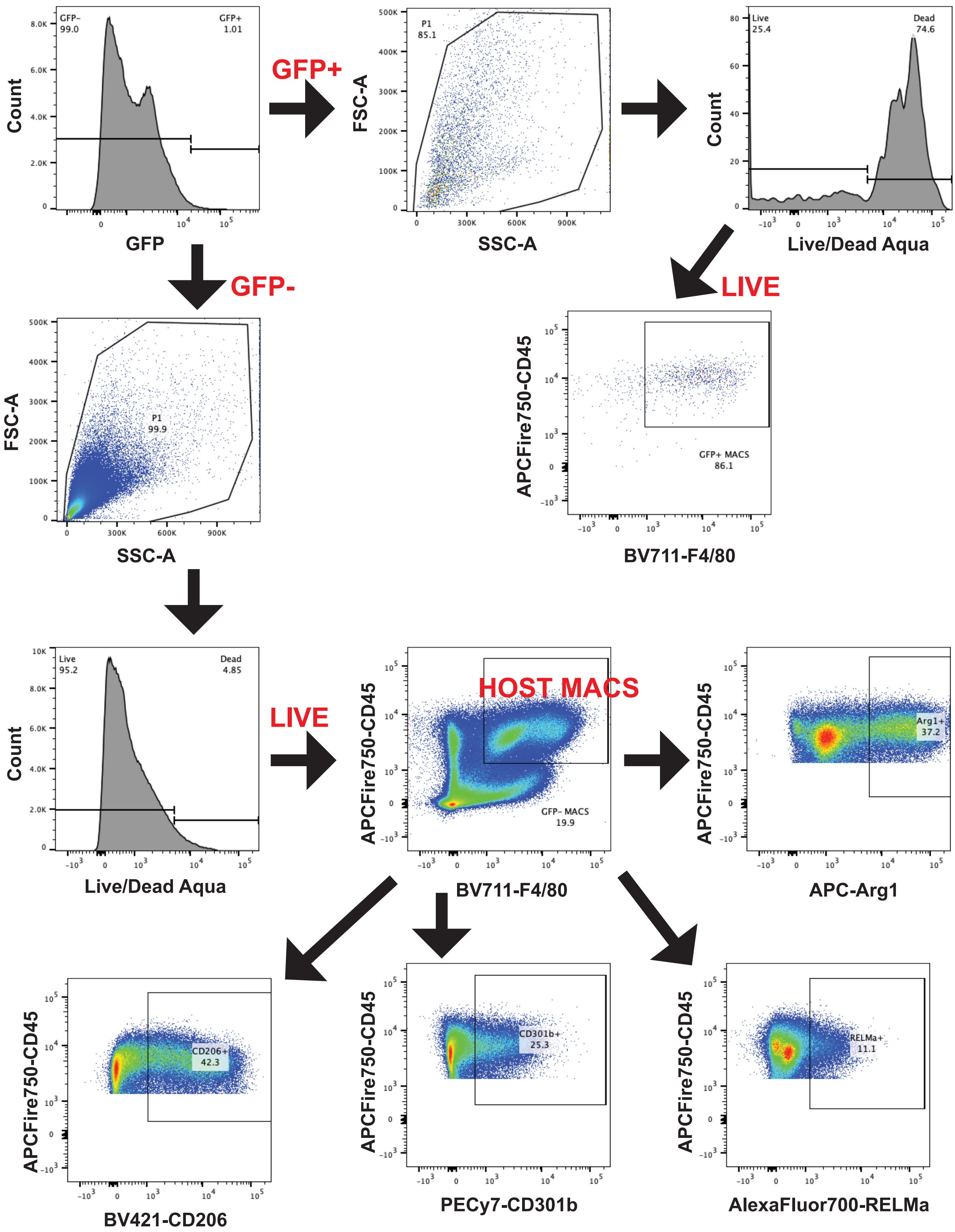
GFP- 99.0
GFP+ 1.01
Count
GFP
GFP+
P1 85.1
FSC-A
SSC-A
Live 25.4
Dead 74.6
Count
Live/Dead Aqua
GFP-
LIVE
GFP+ MACS 86.1
APCFire750-CD45
BV711-F4/80
P1 99.9
FSC-A
SSC-A
Live 95.2
Dead 4.85
Count
Live/Dead Aqua
LIVE
HOST MACS
GFP- MACS 19.9
APCFire750-CD45
BV711-F4/80
Arg1+ 37.2
APCFire750-CD45
APC-Arg1
CD206+ 42.3
APCFire750-CD45
BV421-CD206
CD301b+ 25.3
APCFire750-CD45
PECy7-CD301b
RELMa+ 11.1
APCFire750-CD45
AlexaFluor700-RELMa

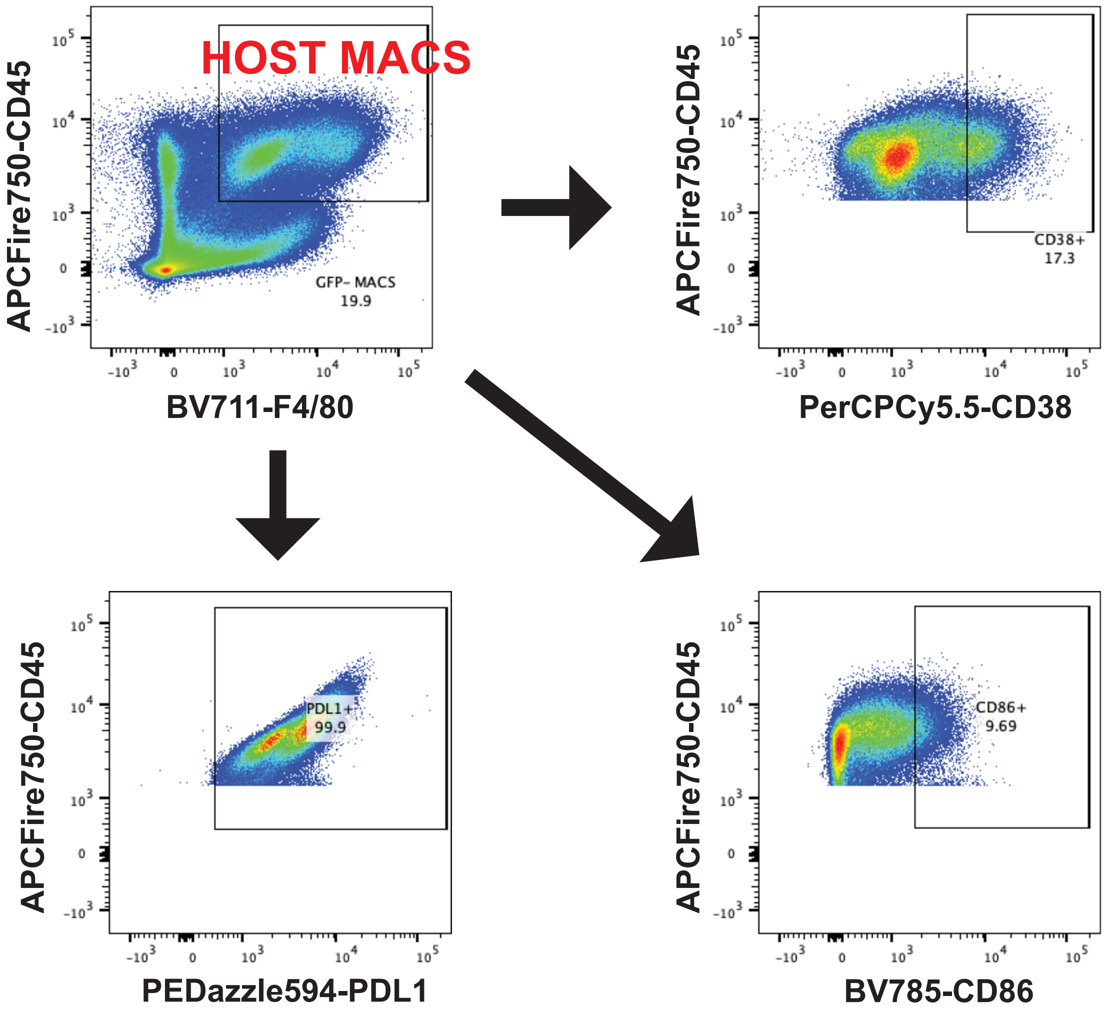

Supplementary Figure 17. Gating strategy for phenotyping of macrophages in mouse injured VML muscle.

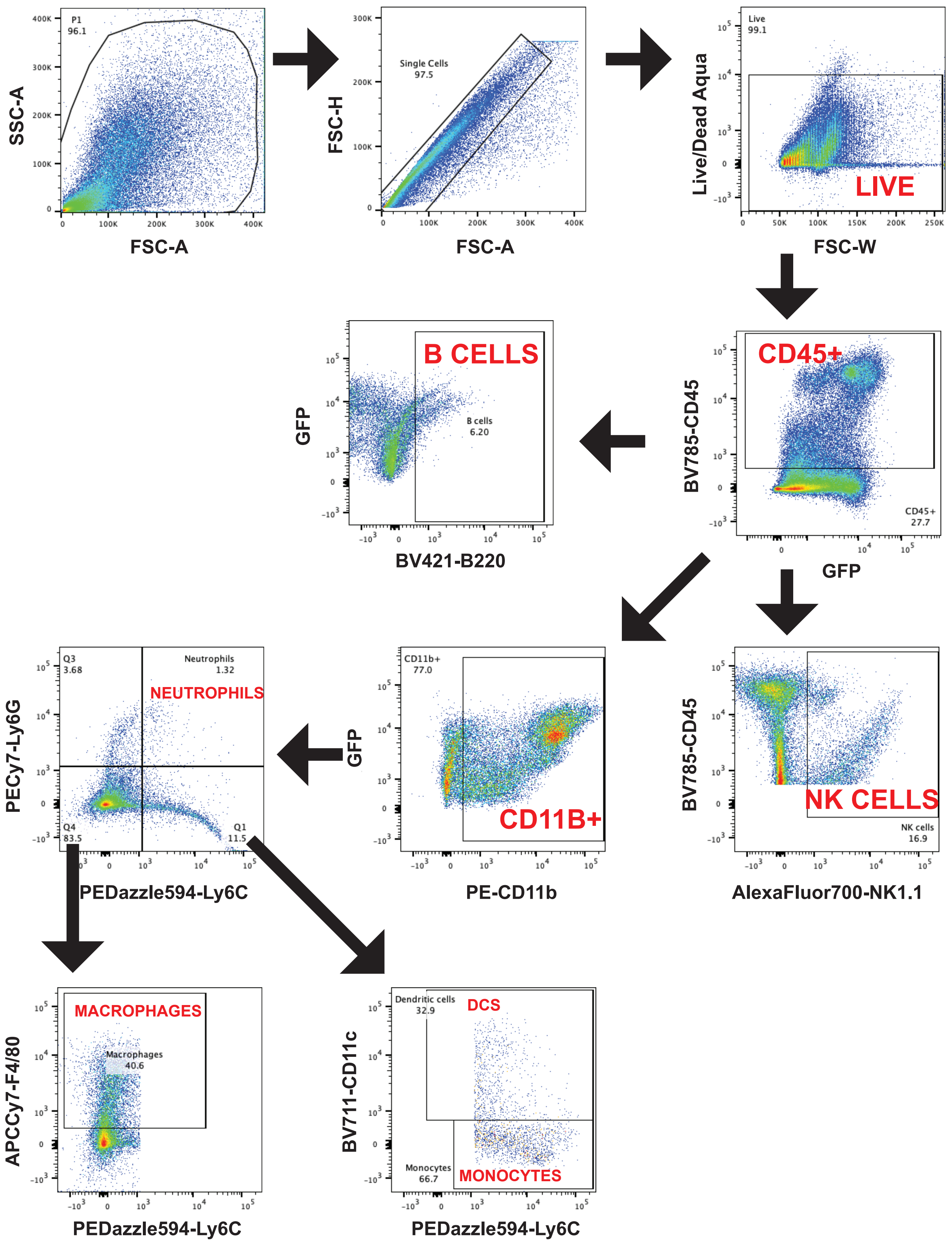

Supplementary Figure 18. Gating strategy for leukocyte classification in mouse injured VML muscle.

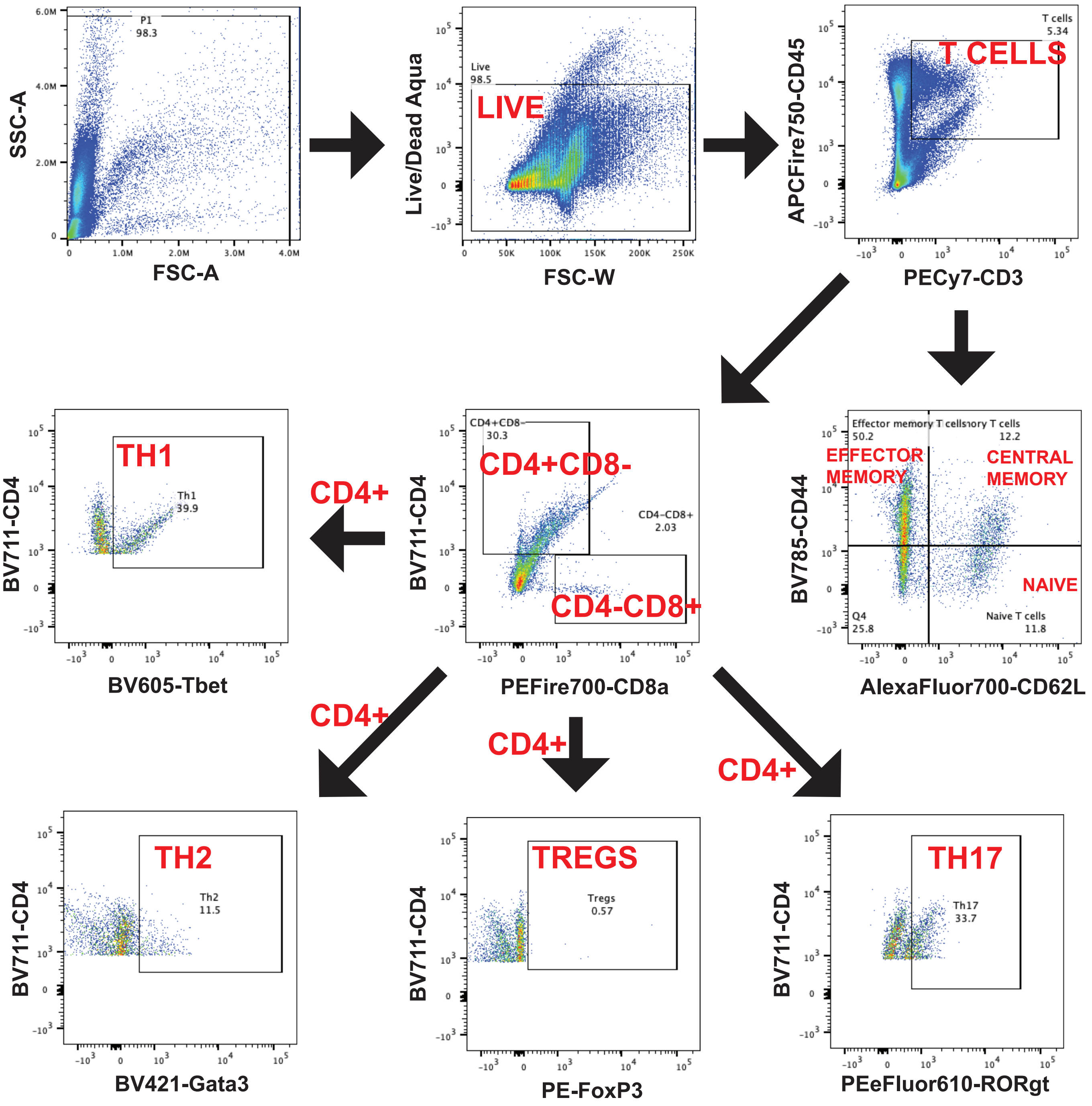

Supplementary Figure 19. Gating strategy for phenotyping of T cell in injured muscle and draining lymph node.

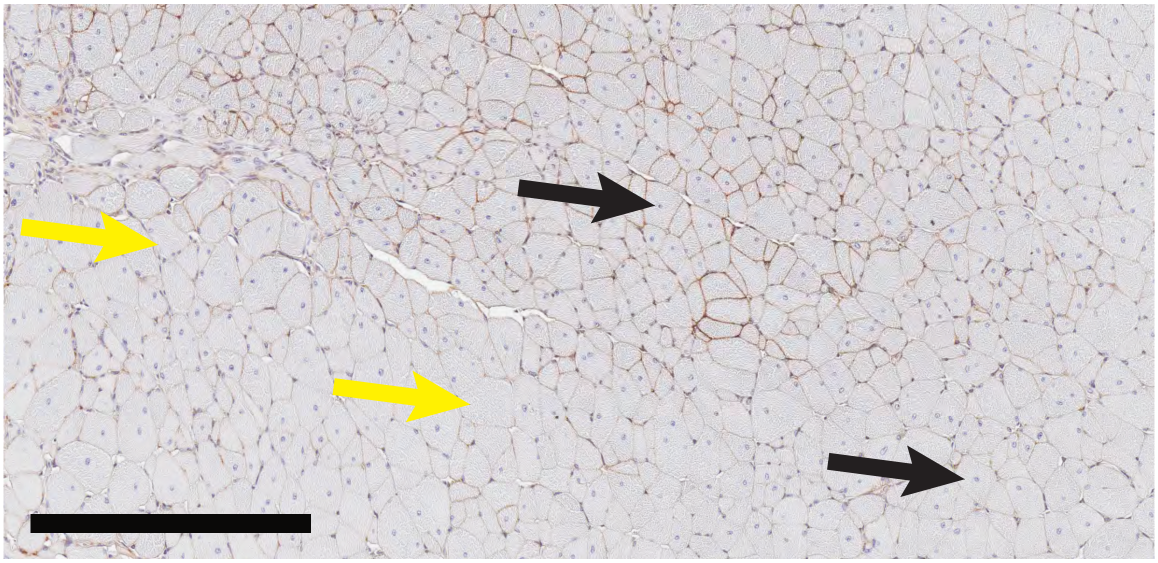

Supplementary Figure 20. Representative image of muscle fibers outlined by laminin stain. Black arrows point to centrally-nucleated (i.e. actively repairing) fibers, while yellow arrows point to peripherally-nucleated or non-nucleated fibers. Scale bar=300μm.
